# Fibrinolytic and Anti-Abdominal Adhesion Effects of Tannin Derivatives from *Rumex nepalensis* Spreng.: Activity-Guided Isolation and Evaluation in a Postoperative Rat Model

**DOI:** 10.1021/acsomega.5c08121

**Published:** 2025-12-05

**Authors:** Gizem Deynez, İpek Süntar, Mürşide Ayşe Demirel, Saadet Özen Akarca Dizakar, Vahap Murat Kutluay, Ece Salihoğlu, Ayşe Kuruüzüm Uz, Osman Tugay

**Affiliations:** a General Directorate of Public Hospitals, Ministry of Health, 06800 Çankaya, Ankara, Türkiye; b Health Sciences Institute, 37511 Gazi University, 06540 Çankaya, Ankara, Türkiye; c Department of Pharmacognosy, Faculty of Pharmacy, 37511 Gazi University, 06330 Yenimahalle, Ankara, Türkiye; d Department of Basic Pharmaceutical Sciences, Faculty of Pharmacy, Gazi University, 06330 Yenimahalle, Ankara, Türkiye; e Department of Histology and Embryology, Faculty of Medicine, Bakırçay University, 35665 Menemen, İzmir, Türkiye; f Department of Pharmacognosy, Faculty of Pharmacy, 37515 Hacettepe University, 06100 Çankaya, Ankara, Türkiye; g Department of Biochemistry, Faculty of Pharmacy, Gazi University, 06330 Yenimahalle, Ankara, Türkiye; h Department of Pharmaceutical Botany, Faculty of Pharmacy, Selçuk University, 42130 Selçuklu, Konya Türkiye

## Abstract

Abdominal adhesions following surgical procedures are a significant cause of postoperative complications, including chronic pain, infertility, and intestinal obstruction. Preventing adhesion formation remains a critical focus of preclinical and clinical research. In this study, we evaluated the fibrinolytic and antiadhesive potential of extracts from four plant species: *Asphodeline lutea* (L.) Rchb., *Rheum ribes* L., *Rubia tinctorum* L., and *Rumex nepalensis* Spreng. Among these, *R. nepalensis* exhibited the highest fibrinolytic activity *in vitro*, and bioactivity-guided fractionation led to the isolation of two major compounds, cinnamtannin B1 (RN2) and epicatechin gallate (RN3). *In vitro* assays demonstrated that RN2 displayed stronger fibrinolytic activity than RN3. The most active extracts, fractions and compound were further evaluated in a rat model of surgically induced intra-abdominal adhesions. A subfraction containing both compounds significantly reduced adhesion formation. These findings indicate that the bioactive constituents of *R. nepalensis*, and potentially the other studied species, may contribute to developing novel antiadhesive therapies. Overall, the study highlights the therapeutic potential of plant-derived flavonoids, anthraquinones, and tannins in adhesion prevention and supports further preclinical investigations to optimize dosage, formulation, and delivery methods. These results provide a foundation for translating natural compounds into practical strategies to reduce postoperative adhesion-related complications.

## Graphical abstract

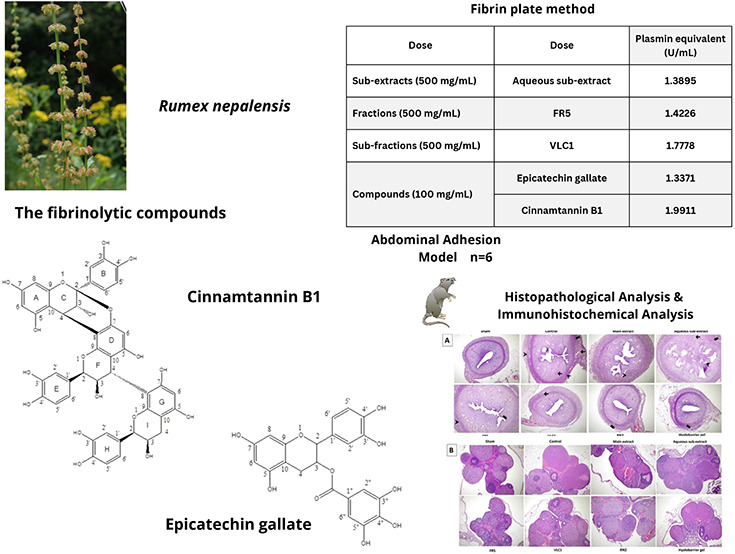

## 1 Introduction

The drug discovery and development process encompasses investigating bioactive compounds derived from natural sources and synthesizing potential pharmacologically active molecules. In recent years, research on medicinal plants has garnered increasing attention, primarily due to the therapeutic potential of plant secondary metabolites. These compounds can serve as direct drug candidates and lead structures for synthesizing bioactive agents.

Intra-abdominal adhesion is a pathological condition resulting from injury to internal organs or the peritoneum, commonly caused by surgical interventions, trauma, burns, foreign bodies, tissue ischemia, or infection. It results from fibrin accumulation due to impaired fibrinolytic activity. The balance between fibrin deposition and degradation plays a key role in regulating routine peritoneal healing and preventing adhesion development.^1,2^ Among the surgical interventions, prior abdominal surgeries, including colorectal, gastrointestinal, gynecological, appendectomy, and cesarean procedures, are recognized as the leading cause. Postoperative adhesions can also result in serious complications, occasionally necessitating further surgical intervention. Due to the limitations of current diagnostic imaging techniques (plain X-rays, water-soluble contrast studies, CT scans, ultrasound and MRI) adhesions are challenging to detect preoperatively.^3^ Moreover, there is currently no definitive or consistently effective treatment modality. Hence, therapeutic measures are typically pursued only after the onset of clinical symptoms. Consequently, the prevention of adhesions and the implementation of prophylactic interventions during surgery are of greater importance than postoperative treatment. Despite efforts to minimize adhesion formation, intra-abdominal adhesions continue to pose a significant clinical challenge and impose a substantial financial burden on healthcare systems, highlighting the insufficiency of current preventive and therapeutic strategies and the need for further scientific investigation.^4,5^

The pathophysiology of adhesion formation involves complex and multifaceted biological processes, including inflammation, oxidative stress, coagulation, fibrinolysis, and cellular proliferation. Given the multifactorial nature of this condition, the structural diversity and multimechanistic bioactivity of secondary metabolites from medicinal plants may offer promising avenues for therapeutic innovation. As a result, interest in medicinal plants’ phytochemicals and biological activities has grown significantly, mainly because of their recognized value in helping to prevent and treat various diseases.^6^

Studies have demonstrated that several phytochemical classes, particularly flavonoids, tannins, and anthraquinones, exhibit anticoagulant, thrombolytic, and fibrinolytic activities, alongside antioxidant and anti-inflammatory properties. These effects suggest that such compounds may hold potential for the prevention and treatment of intra-abdominal adhesions.^7–9^

In the present study, plant species belonging to the same taxonomic groups as those previously identified for their bioactive properties and rich in potentially antiadhesive compounds were selected for evaluation. Methanol (MeOH) extracts prepared from the roots of *Asphodeline lutea* Rchb. (Asphodelaceae), *Rheum ribes* L. (Polygonaceae), *Rubia tinctorum* L. (Rubiaceae), and *Rumex nepalensis* Spreng. (Polygonaceae) were assessed using both *in vitro* (fibrin plate assay) and *in vivo* (experimental intra-abdominal adhesion model in rats) approaches. The fibrinolytic activity of each extract was comparatively evaluated using *in vitro* methods. The plant extract demonstrating the highest efficacy was subsequently selected for activity-guided fractionation. The bioactive compounds responsible for the observed effects were isolated and structurally characterized, and their preclinical efficacy was further examined in the *in vivo* experimental model.

## 2 Materials and Methods

### 2.1 Plant Materials

*Asphodeline lutea* Rchb., *Rheum ribes* L., *Rubia tinctorum* L., and *Rumex nepalensis* Spreng. were collected from their natural habitats. Voucher specimens of each plant were deposited in the Herbarium of the Faculty of Pharmacy at Selçuk University (Table 1). The taxonomic identification of the plant species was performed by Prof. Dr. Osman Tugay from the Department of Pharmaceutical Botany, Faculty of Pharmacy, Selçuk University.

**Table 1:** Collecting Locations and Herbarium Numbers of the Plant Materials

Plant names	Location	Herbarium number
*Asphodeline lutea* (L.) Rchb.	Seydişehir, Toroslar, Konya, Türkiye	KNYA 30195
*Rheum ribes* L.	Islahiye, Amanos Dağı, Gaziantep, Türkiye	KNYA 30192
*Rubia tinctorum* L.	Çiçek Dağı, Kırşehir, Türkiye	KNYA 30193
*Rumex nepalensis* Spreng.	Abant Kuzey yamaçlar, Bolu, Türkiye	KNYA 30194

### 2.2 Extraction of the Plant Materials

The roots of the plants were separated and cut into small pieces. They were then dried at room temperature in a shaded environment and subsequently stored in a cool and dry place. The dried root materials were ground using mechanical mills. Each sample (100 g) was extracted by maceration with 500 mL of MeOH for 72 h at room temperature. The plant residues were subjected to repeated extraction with fresh MeOH under the same conditions. Following each extraction, the macerates were filtered through filter paper, and the combined filtrates were concentrated to dryness under reduced pressure using a rotary evaporator at 40 °C. This extraction process was repeated four times for each plant species. The yields of MeOH extracts from *A. lutea*, *R. ribes*, *R. tinctorum*, and *R. nepalensis* were calculated as 45.084%, 29.103%, 31.583%, and 25.343%, respectively.

### 2.3 Phytochemical Studies on *Rumex nepalensis*

A total of 5.120 g of *Rumex nepalensis* root extract was weighed and dissolved in 100 mL of MeOH. The solution was subjected to liquid–liquid extraction using a separatory funnel, with 100 mL of *n*-hexane applied 20 times. The remaining methanolic phase was then concentrated to dryness under reduced pressure using a rotary evaporator. The resulting residue was dissolved in 100 mL of water and further extracted with dichloromethane (DCM) using a separatory funnel. The *n*-hexane, DCM, and residual aqueous fractions were each concentrated to dryness under reduced pressure at 40 °C using a rotary evaporator.

The aqueous extract (3.261 g) was subjected to column chromatography using Sephadex LH-20 (column dimensions: 3 × 70 cm). The yield of the aqueous extract was calculated as 70.137% (3.591 g). Elution was performed with MeOH, and five fractions were collected—FR1 (0.288 g), FR2 (0.959 g), FR3 (0.239 g), FR4 (0.225 g), and FR5 (0.511 g)—based on phytochemical profile of the compound groups, as determined by normal-phase silica gel thin-layer chromatography (TLC) using the mobile phase system CHCl_3_:MeOH:H_2_O (61:32:7).

To further separate the FR5 fraction (0.474 g), vacuum liquid chromatography (VLC) was conducted using a reverse-phase silica gel stationary phase (column dimensions: 2 × 35 cm). Gradient elution was performed with increasing nonpolarity from water to MeOH using the following solvent systems (all 50% v/v): H_2_O; H_2_O:MeOH (90:10); H_2_O:MeOH (80:20); H_2_O:MeOH (70:30); H_2_O:MeOH (30:20); H_2_O:MeOH (20:30); H_2_O:MeOH (30:70); H_2_O:MeOH (20:80); and MeOH (100). This process yielded two subfractions: VLC1 (0.365 g) and VLC2 (0.030 g).

VLC1 (0.290 g) was dissolved in 5 mL of MeOH and subjected to further purification using Sephadex LH-20 column chromatography, eluted with MeOH. The collected fractions were monitored via TLC (CHCl_3_:MeOH:H_2_O, 65:35:10), combined based on similar chromatographic characteristics, and concentrated into 12 subfractions (FRa-FRl).

The FRh fraction was further purified using preparative thin-layer chromatography (Prep TLC) with the solvent system CHCl_3_:MeOH:H_2_O (65:35:10). Two pure compounds, encoded RN2 (11.3 mg) and RN3 (5.1 mg), were isolated. To increase the amount of RN2, the FRg fraction was subjected to silica gel column chromatography (2 × 70 cm, Kieselgel 60, particle size 0.063–0.2 mm, Merck 7734). Elution was performed with solvent systems of increasing polarity: CHCl_3_:MeOH (90:10, 100 mL); CHCl_3_:MeOH (85:15, 100 mL); CHCl_3_:MeOH:H_2_O (80:20:2, 102 mL); CHCl_3_:MeOH:H_2_O (75:25:2.5, 102.5 mL); CHCl_3_:MeOH:H_2_O (70:30:3, 103 mL); and CHCl_3_:MeOH:H_2_O (65:35:1.5, 115 mL). Fractions were analyzed by TLC (CHCl_3_:MeOH:H_2_O, 65:35:10) and combined into five subfractions based on similarity in polarity and compound profile. From this procedure, compound RN2 was obtained in pure form (21.5 mg).

Structural elucidation of the RN2 and RN3 compounds was performed using spectroscopic techniques, including one-dimensional (1D) and two-dimensional (2D) nuclear magnetic resonance (NMR), mass spectrometry (MS), and high-resolution mass spectrometry (HR-MS).

### 2.4 *In Vitro* Activity Studies

#### 2.4.1 Fibrin Plate Method

Fibrinolytic activity was assessed using a slightly altered version of the fibrin plate method.^10,11^ Fibrinolytic activity was assessed by measuring the clear zones formed due to fibrin degradation, and the lysis area is determined by calculating the diameters of the lysis zones (Figure 1). For this aim, bovine fibrinogen and thrombin were added to the 1% agarose mixture dissolved in 50 mM pH 7.8 Tris-HCl buffer before it solidified completely. It was poured into a glass Petri dish and incubated at 37 °C for 1 h. At the end of incubation, at least six holes with a diameter of 4 mm were opened in the resulting fibrin clot layer. Test samples are applied onto the surface of the fibrin layer, and the plates are then incubated at 37 °C under humid conditions for 24 h.

**Figure 1:**
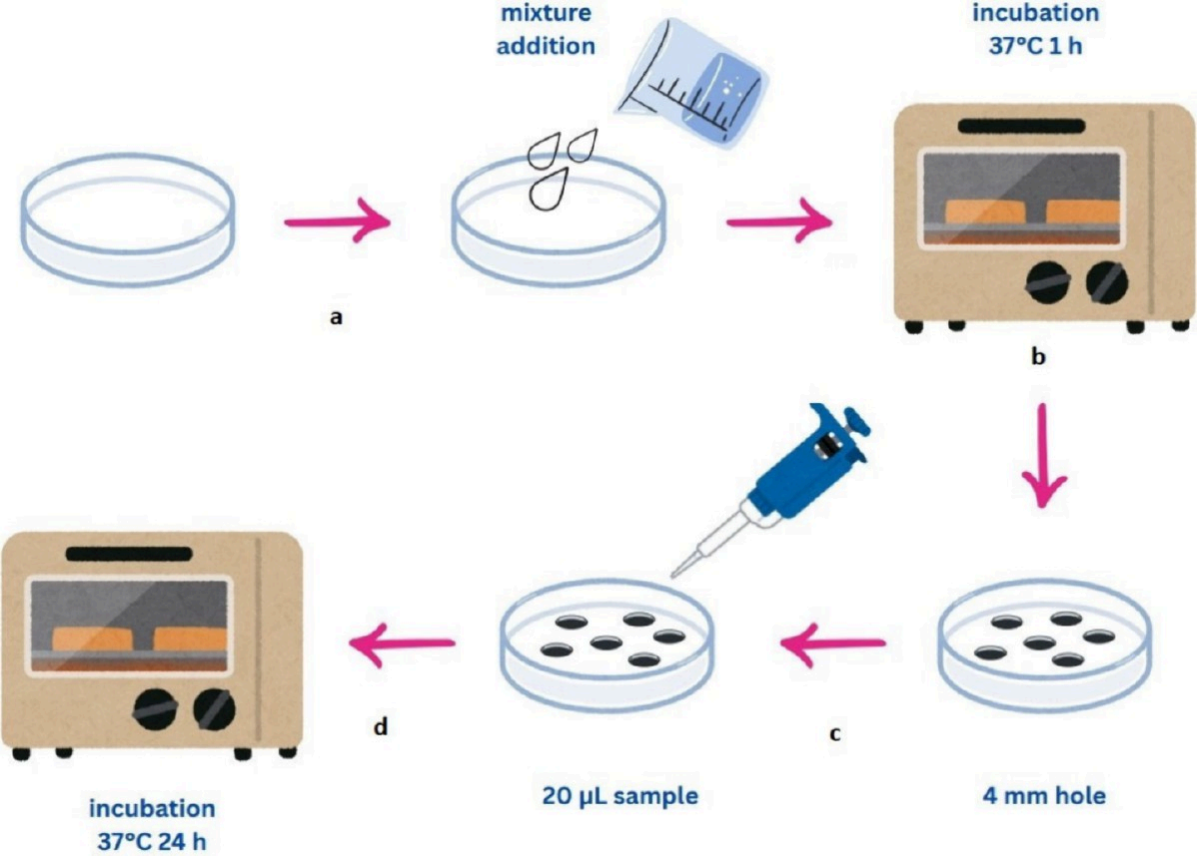
Procedural steps of the fibrin plate method: a) preparation of the fibrin clot; b) incubation; c) application of test samples; d) incubation and observation of lysis zones.

Samples were added to the wells opened in the Petri dish in a volume of 20 μL. The Petri dish was incubated for 24 h at 37 °C. At the end of incubation, two perpendicular diameters of the lysis zones in the Petri dish were measured, and the lysis area (mm^2^) was calculated using the formula “π x r_1_ x r_2_”. A standard curve was constructed by plotting the lysis area of plasmin against its concentration. The areas were converted into concentrations through interpolation on a plasmin reference curve.

### 2.5 *In Vivo* Activity Studies

Ten-week-old female, healthy, 48 Wistar Albino rats weighing 200–250 g were purchased from Gazi University Laboratory Animal Breeding and Experimental Research Center, Ankara, Türkiye. The study was conducted under the ARRIVE (Animal Research: Reporting of *in vivo* Experiments) guidelines. All experimental procedures were approved by the Experimental Animal Ethics Committee of Gazi University (G.Ü.ET-22.015). The animals were kept in polysulfone cages with aspen shavings for bedding at 21–24 °C, 40–45% humidity, and light-controlled (12 h light/12 h dark) conditions at the Laboratory Animal Care and Research Unit, Faculty of Pharmacy, Gazi University (Ankara, Turkiye). All rats were quarantined for 1 week before the experimental procedure. Rats were fed a standard pellet diet and tap water ad libitum throughout the experimental period. Following a one-week acclimatization period, all animals included in the study for the induction of the intra-abdominal adhesion model were randomly assigned to groups, with six rats per group (Figure 2).

**Figure 2:**
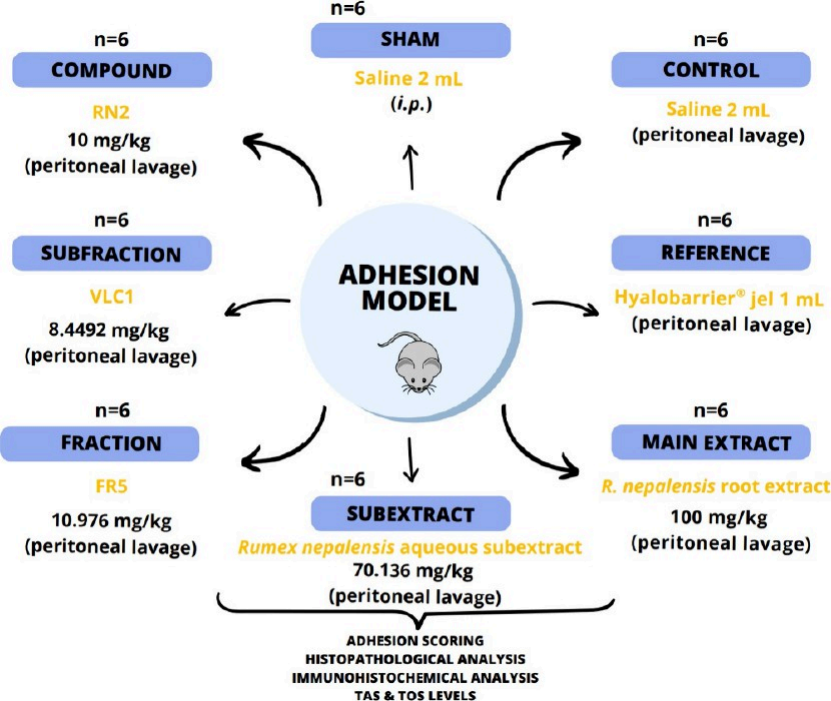
Experimental group on abdominal adhesion in rats. Note that the abdominal adhesion model was created in all animals except the sham group.

#### 2.5.1 Surgically Induced Postoperative Adhesions in Rats

Before the surgical procedure, the rats were anesthetized using intraperitoneal (*i.p*.) administration of xylazine (8 mg/kg) and ketamine (75 mg/kg). The animals were placed in the supine position, and the abdominal hair was shaved and disinfected with povidone-iodine. Subsequently, an approximately 3 cm incision was created along the caudal ventral midline to expose the peritoneal cavity, and both uterine horns were exteriorized. To induce the abdominal adhesion model, petechial hemorrhages were generated in each uterine horn using a no. 15 scalpel blade. Similarly, traumatic lesions were created on the abdominal wall in alignment with the uterine horns using the same scalpel technique.^12^ The uterine adhesion model was induced in all animals except those in the sham group.

#### 2.5.2 Treatment Procedure

All test materials were administered *i.p*. in a single dose before closing the abdominal cavity. The sham and control groups were given 1 mL of saline. The reference group was treated with 1 mL of Hyalobarrier gel.^13^ The main extract was administered to the experimental animals at a dose of 100 mg/kg. Dose–effect evaluations were subsequently conducted for the subextract, fraction, and subfraction. Based on previously published studies, the administration dose for the isolated compound was determined to be 10 mg/kg.^14–17^ Following the procedure, the abdominal muscle and skin layers were sutured using USP 3/0 polyglactin (Lactasorb PGLA, Orhan Boz, Türkiye).

#### 2.5.3 Termination of Experimental Procedure

All the rats were sacrificed using the exsanguination method under general anesthesia (8 mg/kg i.p. xylazine hydrochloride and 75 mg/kg i.p. ketamine hydrochloride) 14 days after the adhesion model was induced. The adhesion scores were evaluated according to the scoring system presented in Table 2, which was based on the criteria described by Leach et al. (1998).^74^ Subsequently, the uterine and ovarian tissues were collected for histopathological analyses.

**Table 2:** Adhesion Scoring

Degree	According to the defect in the uterine cornua	Adhesion severity in macroscopic examination	According to the degree of adhesion
0	No adhesion	No adhesion	No adhesion
1	1–25% adhesion area	Thin avascular	Adhesion that separates with a light touch
2	26–50% adhesion area	Vascular or opaque	Adhesion that separates with moderate traction
3	51–75% adhesion area	Adherent junctions	Adhesion that requires dissection
4	76–100% adhesion area	-	-

### 2.6 Histopathological Analysis

Tissue samples collected from adhesion sites, the cornu uteri, and ovaries of rats were fixed in 10% buffered formalin and embedded in paraffin. Sections of 4 μm thickness were obtained from the paraffin blocks following standard histological procedures. These sections were stained with hematoxylin and eosin (H&E) and Masson’s Trichrome and examined under a light microscope (Zeiss Axioscope 5, Germany). Histopathological evaluation focused on assessing the severity of inflammatory changes and fibrosis in the cornu uteri and recording stromal and parenchymal alterations in the ovarian tissue.^18^ The extent of fibrosis was quantified by calculating the percentage of blue-stained collagen fiber areas in Masson’s Trichrome-stained sections using the ImageJ software.^19^

#### 2.6.1 Hematoxylin & Eosin (H&E) Staining Protocol

Paraffin-embedded tissue sections, four μm in thickness, were prepared following standard histological procedures. After deparaffinization, the sections were rehydrated by immersion in a graded series of ethanol solutions (100%, 90%, 80%, 70%, and 50%) for 10 min each. After air-drying, the slides were rinsed under running tap water for 10 min to remove residual alcohol. Subsequently, the sections were stained with Harris hematoxylin for 10 min and rinsed under running tap water for another 10 min. Differentiation was achieved by briefly dipping the sections 2–3 times in a solution composed of glacial acetic acid and ethanol, followed by another rinse under running water for 10 min. The sections were then stained with eosin for 10 min, rewashed under running water for 10 min, and dehydrated through a graded ethanol series (50%, 70%, 80%, 90%, and 100%). Following dehydration, the slides were cleared in xylene for 45 min and mounted using Entellan. Histological evaluation of the stained sections was conducted using a ZEISS Axioscope 5 computer-assisted light microscope.

#### 2.6.2 Masson’s Trichrome Staining Protocol

Masson’s Trichrome staining was applied to 4 μm-thick tissue sections to assess fibrotic changes in the adhesion sites of the experimental groups. Following staining, the sections were mounted using a suitable mounting medium with a coverslip and examined under a ZEISS Axioscope 5 computer-assisted light microscope. The extent of fibrosis was quantified by calculating the percentage of blue-stained collagen fiber areas using the ImageJ software.^19^

### 2.7 Immunohistochemical Analysis

Paraffin-embedded tissue sections were deparaffinized, rehydrated, and rinsed with phosphate-buffered saline (PBS) (pH 7.4). For antigen retrieval, the sections were treated with citrate buffer (pH 6.0) and subsequently rinsed with distilled water. Endogenous peroxidase activity was blocked by treating the tissue samples with 3% hydrogen peroxide, followed by a 5 min rinse in PBS. To prevent nonspecific binding, the UltraV block was applied. The sections were then incubated with primary antibodies against tumor necrosis factor (TNF)-α, interleukin (IL)-6, and IL-8 (diluted 1:100; Elabscience) and washed three times with PBS. A biotin-conjugated secondary antibody was applied for 10 min, followed by a 10 min incubation with a streptavidin-peroxidase enzyme complex. Immunoreactivity was visualized by adding diaminobenzidine (DAB) chromogen and counterstaining with Mayer’s hematoxylin. After dehydration through a graded alcohol series, clearing in xylene, and mounting with Entellan, the slides were examined at × 400 magnification using a ZEISS Axioscope 5 microscope. Immunopositivity for TNF-α, IL-6, and IL-8 was quantified as a percentage using ImageJ analysis across six randomly selected fields for each group.

### 2.8 Statistical Analysis

Statistical analyses were performed using GraphPad Prism 8.0. Values were expressed as the mean ± standard error of the mean (SEM). Differences between groups were analyzed using a one-way analysis of variance (ANOVA). A value of *p* < 0.05 was considered statistically significant.

## 3 Results

### 3.1 Phytochemical Analysis

By bioassay-guided fractionation and isolation, two compounds (RN2 and RN3) were isolated from the MeOH extract of the root parts of the *R. nepalensi*s. The structural analysis of the compounds was conducted using spectroscopic methods, including 1D and 2D NMR and HR-MS (see the Supporting Information, Figures S1–S16 and Tables S1 and S2). In the light of all these findings and comparing the results with literature data, cinnamtannin B1 ^20,21^ (RN2) and (−)-epicatechin gallate (RN3) were determined.^22^ However, minor impurities or coeluting constituents cannot be completely excluded.

### 3.2 Fibrinolytic Activity Results of the Extracts

The extracts were dissolved in 50% aqueous ethanol and applied to 4 mm diameter holes in the Petri dishes using a 20 μL micropipette. Each sample was prepared at 125, 250, and 500 mg/mL concentrations and tested in duplicate. The samples were incubated in a drying oven at 37 °C for 24 h. At the end of the incubation period, the width and length diameters of the lysis zones induced by the extracts were measured. The lysis zones of the plant extracts are presented in Table 3. The plasmin equivalent corresponding to the lysis zones of the plant extracts was calculated. The plant species with the highest fibrinolytic activity was determined to be *R. nepalensis* with the 1.3895 U/mL plasmin equivalent value at 500 mg/mL concentration.

**Table 3:** Lysis Zones of the Plant Extracts

Plant material	Concentration (mg/mL)	Average radius (*r*) value (mm)	Average of lysis zones^a^ (mm^2^)	Plasmin equivalent (U/mL)
*Asphodeline lutea*	125	0	-	-
	250	0	-	-
	500	0	-	-
*Rheum ribes*	125	3.000	28.26000	-
	250	5.000	78.50000	-
	500	5.000	78.50000	-
*Rubia tinctorum*	125	3.000	28.26000	-
	250	5.000	78.50000	-
	500	7.500	176.62500	0.6736
*Rumex nepalensis*	125	5.000	78.50000	-
	250	7.500	176.62500	0.6736
	500	12.500	490.62500	1.3895
Control	50% Ethanol–Water	0	-	-

a Since the constant value is 86.841 in the quadratic equation with one unknown, the unit equivalent of those with an area less than 86.841 mm^2^ cannot be calculated.

### 3.3 Fibrinolytic Activity Results of the Subextracts, Fractions, Subfractions, and Compounds

The subextracts were dissolved in 50% aqueous ethanol and applied to 4 mm diameter holes in Petri dishes using a 20 μL micropipette. Each sample was prepared at a 500 mg/mL concentration and tested in duplicate. The samples were then incubated in a drying oven at 37 °C for 24 h. After the incubation period, the diameters of the lysis zones were measured. The resulting lysis zones for the subextracts, fractions, subfractions, and compounds are presented in Figure 3 and Table 4. Among the subextracts, the aqueous subextract (1.3895 U/mL) exhibiting the highest fibrinolytic activity was fractionated using chromatographic methods. Among the fractions, FR5 (1.4226 U/mL) demonstrated the highest fibrinolytic activity, and further fractionation studies were continued with this fraction. Subsequently, isolation studies proceeded with VLC1 (1.7778 U/mL), which showed the highest fibrinolytic activity among the subfractions. The fibrinolytic activities of the isolated compounds were also assessed using *in vitro* methods. The results revealed that RN2 exhibited a significantly higher fibrinolytic effect than RN3 with the 1.9911 U/mL plasmin equivalent value at 500 mg/mL concentration. Based on these findings, RN2 was selected for further *in vivo* investigation to evaluate its efficacy in an intra-abdominal adhesion model.

**Table 4:** Lysis Zones of Subextracts, Fractions, Subfractions, and Compounds of *R. nepalensis*

Dose	Extract/Fraction/Compound	Average radius (*r*) value (mm)	Average of lysis zones^a^ (mm^2^)	Plasmin equivalent (U/mL)
Subextracts (500 mg/mL)	*n*-Hexane subextract	2.750	23.94250	-
	DCM subextract	5.000	78.50000	-
	Aqueous subextract	12.500	490.62500	1.3895
Fractions (500 mg/mL)	FR1	3.000	28.26000	-
	FR2	3.500	38.46500	-
	FR3	4.500	63.58500	-
	FR4	5.000	78.50000	-
	FR5	12.750	510.64250	1.4226
Subfractions (500 mg/mL)	VLC1	15.500	755.17000	1.7778
	VLC2	5.500	94.98500	0.2289
Compounds (100 mg/mL)	RN2	17.125	928.65500	1.9911
	RN3	11.875	460.01000	1.3371
50% Ethanol–water	Control	0	-	-

a Since the constant value is 86.841 in the quadratic equation with one unknown, the unit equivalent of those with an area less than 86.841 mm^2^ cannot be calculated; FR: Fraction; VLC1: Subfraction; RN2: Cinnamtannin B1; RN3: (−)-Epicatechin gallate.

**Figure 3:**
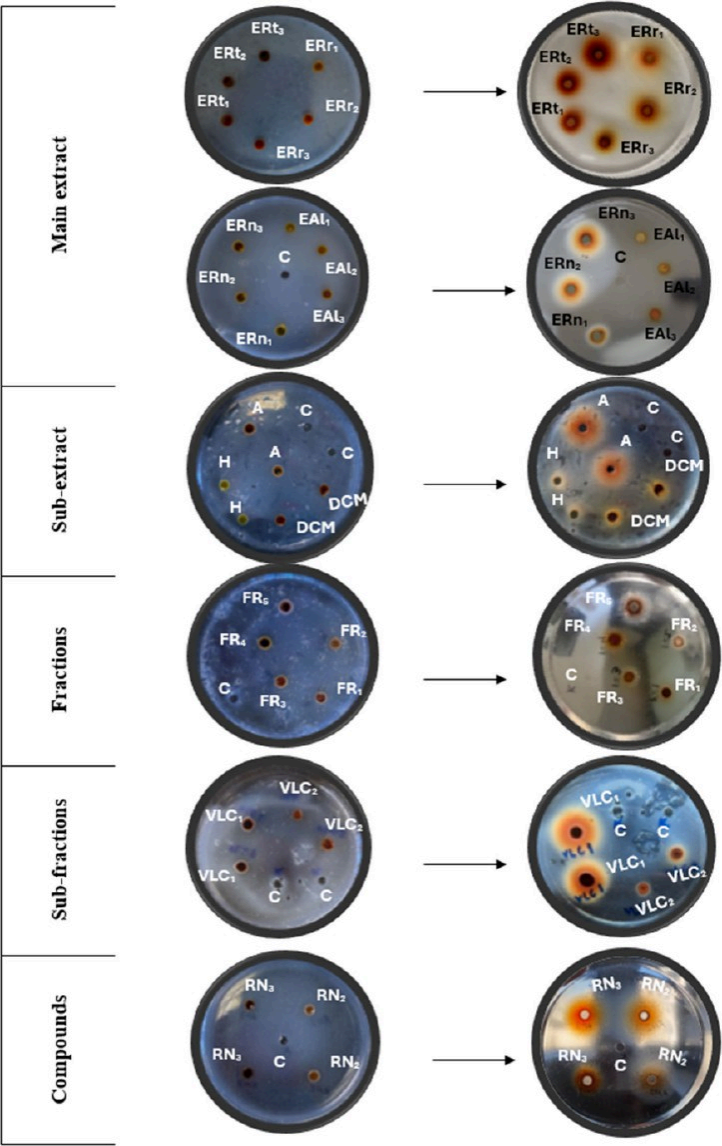
Lysis zones (*Rubia tinctorum* extract; ERt_1_: 125 mg/mL, ERt_2_: 250 mg/mL; ERt_3_: 500 mg/mL; *Rumex nepalensis* extract; ERn_1_: 125 mg/mL, ERn_2_: 250 mg/mL, ERn_3_: 500 mg/mL; *Rheum ribes* extract; ERr_1_: 125 mg/mL, ERr_2_: 250 mg/mL, ERr_3_: 500 mg/mL; *Asphodeline lutea* extract; EAl_1_: 125 mg/mL, EAl_2_: 250 mg/mL, EAl_3_: 500 mg/mL; A: Aqueous, DCM: Dichloromethane, H: *n*-Hexane, FR: Fraction, VLC1: Subfraction, RN2: Cinnamtannin B1, RN3: (−)-Epicatechin gallate, C: Control).

### 3.4 *In Vivo* Biological Activity Findings

The effects of *R. nepalensis* root extract (main extract), aqueous subextract, FR5, VLC1, and RN2 on adhesion formation were evaluated in an experimental intra-abdominal adhesion model in rats (Figure 4).

**Figure 4:**
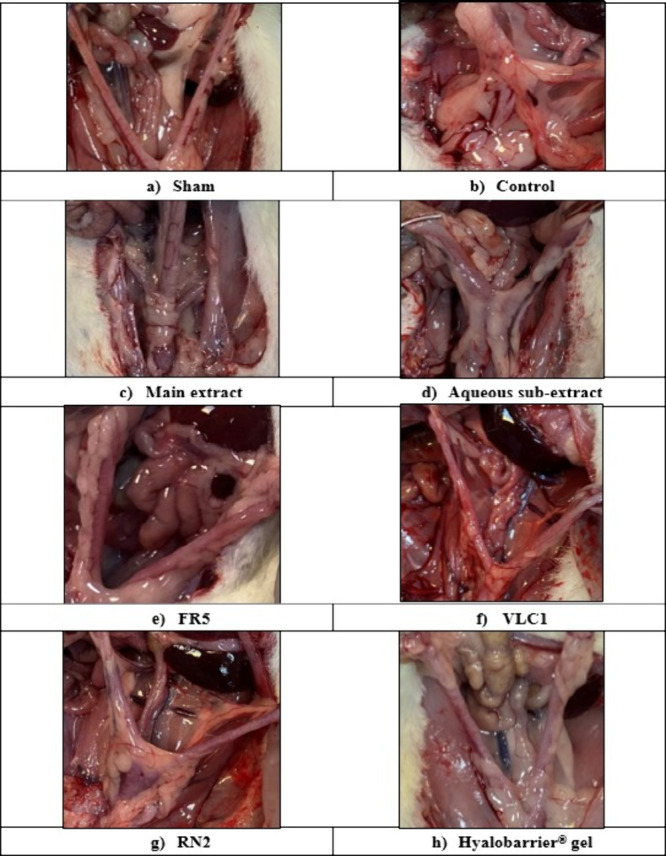
Intra-abdominal adhesion model: a) Sham group; b) Control group; c) Main extract: *Rumex nepalensis* extract; d) Aqueous subextract; e) FR5: Fraction 5; f) VLC1: Subfraction; g) RN2: Cinnamtannin B1; h) Hyalobarrier gel: Reference drug.

Adhesion development in the experimental animals was macroscopically scored based on the extent, severity, and degree of the damage, and the adhesion scores are presented in Table 5. When the scores were analyzed, it was found that all treatment groups, except for the aqueous subextract group, showed statistically significant effectiveness compared to the control group (*p* < 0.0001). The main extract, FR5, VLC1 and RN2 significantly reduced the adhesion formation.

**Table 5:** Adhesion Scores in Intra-Abdominal Adhesion Models according to Experimental Groups^a^

Groups	Score 1	Score 2	Score 3
Sham	0.00 ± 0.00****	0.00 ± 0.00****	0.00 ± 0.00****
Control	4.00 ± 0.00	3.00 ± 0.00	3.00 ± 0.00
Main extract	1.00 ± 0.00****	1.00 ± 0.00****	1.00 ± 0.00****
Aqueous subextract	3.20 ± 0.40	2.50 ± 0.22	2.50 ± 0.22
FR5	1.50 ± 0.22****	1.00 ± 0.00****	0.50 ± 0.22****
VLC1	1.30 ± 0.49****	0.67 ± 0.21****	0.83 ± 0.31****
RN2	2.00 ± 0.26****	1.70 ± 0.33****	1.50 ± 0.22****
Hyalobarrier gel	1.60 ± 0.24****	1.60 ± 0.24****	1.40 ± 0.25****

a Score 1: Evaluation of adhesion according to the defect in the uterine cornua; Score 2: Severity of adhesion in macroscopic examination; Score 3: According to the degree of adhesion; ****: *p* < 0.0001 (results were compared with the control group); Main extract: *Rumex nepalensis* extract; FR5: Fraction 5; VLC1: Subfraction; RN2: Cinnamtannin B1; Hyalobarrier gel: Reference drug.

### 3.5 Histopathological Analysis

When the percentage of fibrotic areas in the uterine tissue sections of the experimental groups was evaluated, the sham group exhibited a statistically significantly lower fibrotic area percentage compared to the control, reference (Hyalobarrier gel), main extract, aqueous subextract, FR5, VLC1, and RN2 groups (respectively; *p* < 0.001; *p* = 0.006; *p* < 0.001; *p* < 0.001; *p* < 0.001; *p* < 0.001). The fibrotic area percentage in the control group was significantly higher than in the reference group (*p* < 0.001). The fibrotic area percentages in the VLC1 and RN2 groups were statistically significantly lower than in the control group (respectively; *p* = 0.026 and *p* = 0.001).

No significant difference was observed among the other groups. When the percentage of fibrotic area in the ovarian tissue sections was compared statistically, the average fibrotic area percentage in the sham group was lower than in the other groups. However, statistical significance was observed only when compared to the VLC1 subfraction group (*p* = 0.026). No significant differences were found between the other groups (Figure 5A and B). As a result, a normal histomorphological appearance was observed in the uterine and ovarian tissue sections of the Sham group. In contrast, the control group exhibited inflammation, congestion, and fat cell infiltration in the uterine sections. Although these changes persisted in some areas in the treatment groups, histopathological alterations were partially reduced, with this effect being most prominent in the VLC1 and RN2 groups (A). In the ovarian tissue, no significant differences were observed in the stroma and parenchyma across the experimental groups (B). The tissues were further evaluated for fibrosis and pro-inflammatory cytokines. Notably, histopathological changes were more pronounced in the uterus than in the ovaries. An anti-inflammatory effect was generally observed in the treatment groups, with a partial therapeutic effect on adhesion formation, particularly in the VLC1 and RN2 groups (Figure 6A and B).

**Figure 5:**
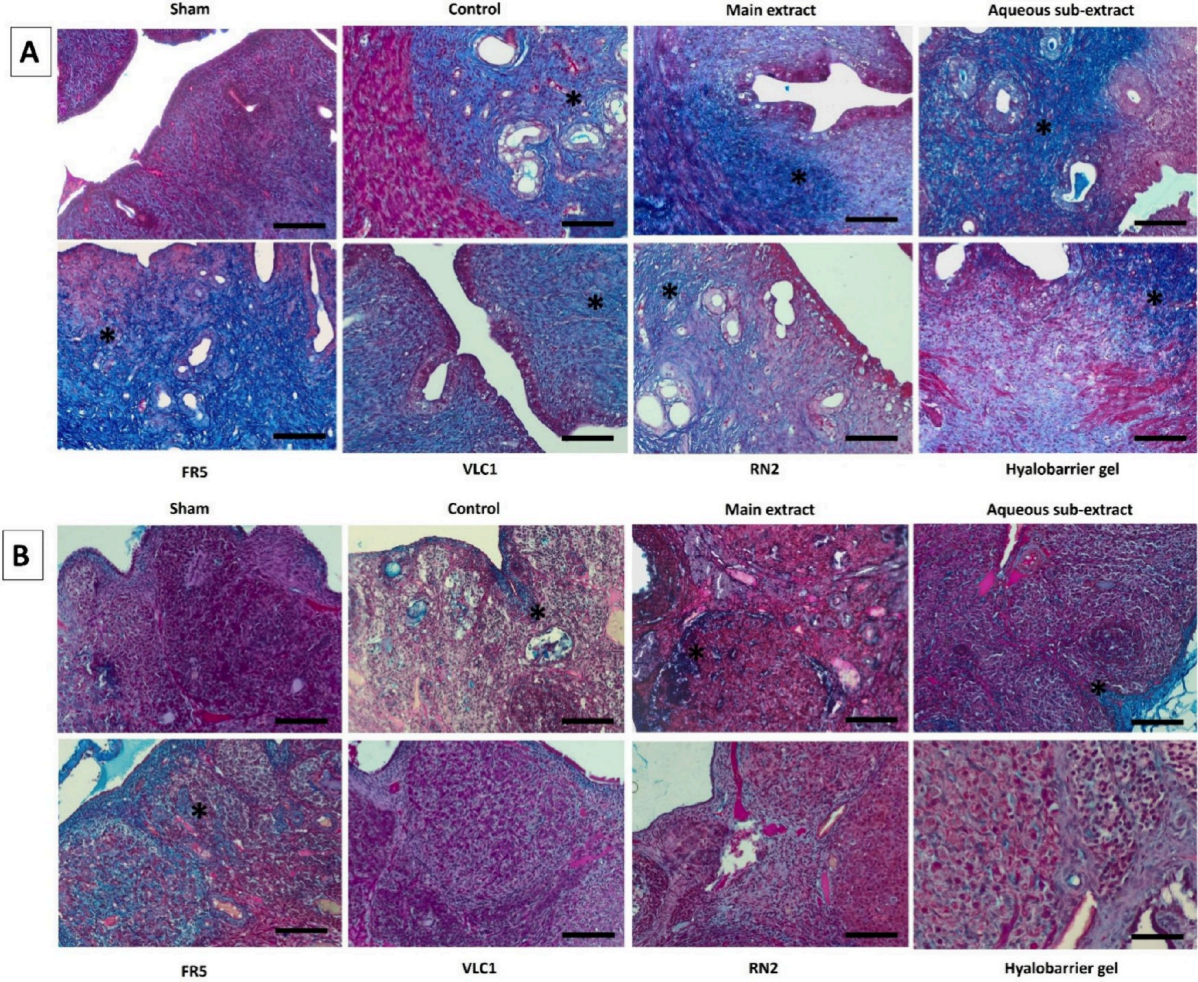
A) Fibrotic areas (*) are seen in the uterine sections of the experimental groups (MT, X400). B) Fibrotic areas (*) are observed in the ovarian sections of the experimental groups (MT, X400 scale bar 50 μm); Main extract: *Rumex nepalensis* extract; FR5: Fraction 5; VLC1: Subfraction; RN2: Cinnamtannin B1; Hyalobarrier gel: Reference drug.

**Figure 6:**
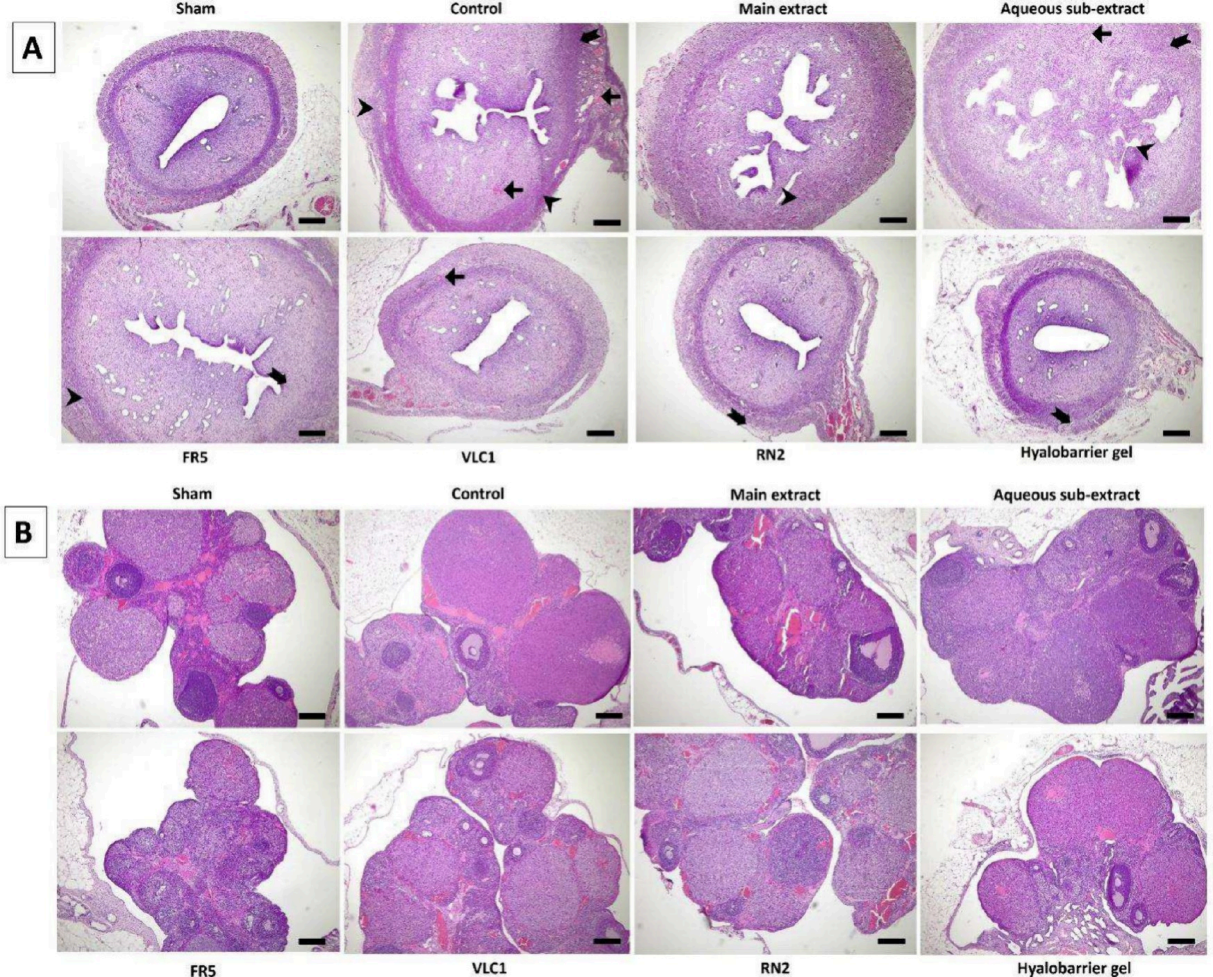
A) Inflammation (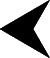), congestion (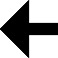), and fibrosis (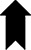) are observed in the uterus sections of experimental groups. B) Ovarian sections of experimental groups (HE, X50 scale bar 200 μm); Main extract: *Rumex nepalensis* extract; FR5: Fraction 5; VLC1: Subfraction; RN2: Cinnamtannin B1; Hyalobarrier gel: Reference drug.

### 3.6 Immunohistochemical Analysis

When the percentages of TNF-α, IL-6, and IL-8 immunopositive areas in the uterine tissue of the experimental groups were evaluated statistically, the percentage of TNF-α immunopositive areas in the Sham group was significantly lower than in the other experimental groups (*p* < 0.001). It was noted that TNF-α immunoreactivity in the RN2 compound group decreased statistically significantly compared to the control, extract, and subextract groups (respectively; *p* = 0.021; *p* = 0.045; *p* = 0.041).

Regarding IL-6, the percentages of immunopositive areas in the uterine tissue of the experimental groups were statistically evaluated. The percentage of IL-6 immunopositive areas in the Sham group was significantly lower than in the other experimental groups (respectively; *p* = 0.02; *p* < 0.001; *p* < 0.001; *p* < 0.001; *p* < 0.001; *p* < 0.001; *p* < 0.001). The IL-6 immunopositive areas in the control, aqueous subextract, and fraction (FR5) groups were significantly higher than in the reference group (respectively; *p* < 0.001; *p* = 0.001; *p* = 0.019). Furthermore, the percentages of IL-6 immunopositive areas in the main extract and subfraction (VLC1) groups were significantly lower compared to the control group (respectively; *p* = 0.024; *p* = 0.005).

When evaluating the percentage of IL-8 immunopositive areas in the experimental groups, no statistically significant differences were observed between the groups. However, the lowest percentage of immunoreactivity among the treatment groups was seen in the RN2 group. A decrease in IL-8 immunoreactivity was also noted in the control group, though it was not statistically significant (Table 6 and Figure 7).

**Table 6:** Uterus Immunopositive Area Percentages^a^

Groups	Fibrotic area (%)	TNF-α immunopositive area percentage (%)	IL-6 immunopositive area percentage (%)	IL-8 immunopositive area percentage (%)
Sham	16.90 ± 0.60	0.40 ± 0.10	1.41 ± 0.25	0.26 ± 0.05
Control	42.35 ± 2.61	21.36 ± 1.10	20.54 ± 1.13	14.63 ± 0.89
Main extract	33.23 ± 1.90	20.14 ± 0.72	14.98 ± 0.79*	13.43 ± 0.63
Aqueous subextract	32.77 ± 2.18	19.95 ± 0.71	17.57 ± 0.70	13.44 ± 0.56
FR5	30.03 ± 0.79	19.96 ± 0.86	16.81 ± 0.94	14.07 ± 0.70
VLC1	29.69 ± 1.46	19.10 ± 0.69	14.14 ± 0.76**	14.28 ± 0.66
RN2	27.34 ± 1.68	16.30 ± 0.68*	15.59 ± 0.64	12.80 ± 0.66
Hyalobarrier gel	25.38 ± 1.47***	17.55 ± 0.49	12.35 ± 0.62***	12.06 ± 0.53

a *: *p* < 0.05; **: *p* < 0.01; ***: *p* < 0.001 (results were compared with the control group); Main extract: *Rumex nepalensis* extract; FR5: Fraction 5; VLC1: Subfraction; RN2: Cinnamtannin B1; Hyalobarrier gel: Reference drug.

**Figure 7:**
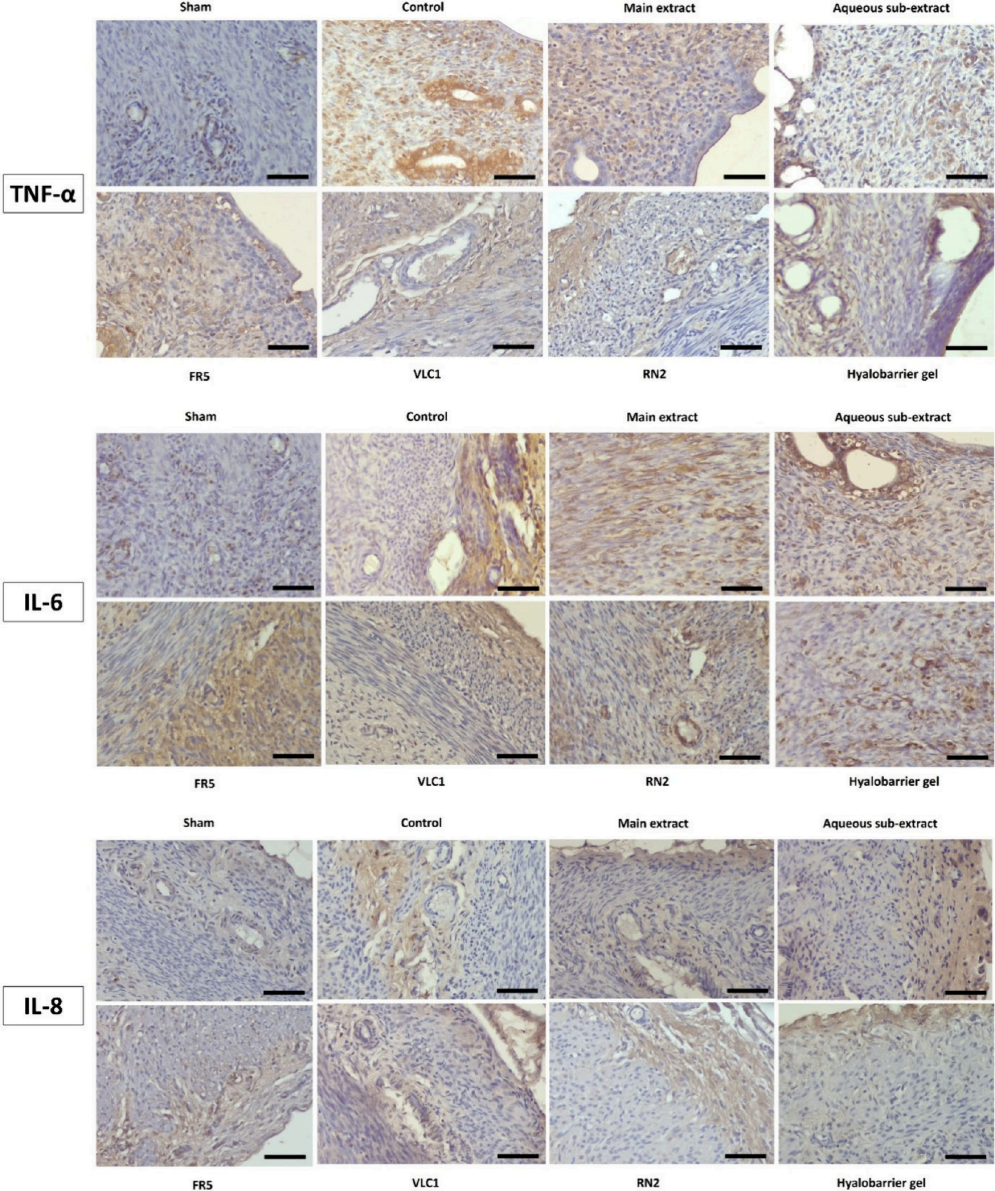
TNF-α: TNF-α immunoreactivity in uterine sections of experimental groups (DAB, Mayer’s hematoxylin, X400). IL-6: IL-6 immunoreactivity in uterine sections of experimental groups (DAB, Mayer’s hematoxylin, X400). IL-8: IL-8 immunoreactivity in uterine sections of experimental groups (DAB, Mayer’s hematoxylin, X400 scale bar 50 μm); Main extract: *Rumex nepalensis* extract; FR5: Fraction 5; VLC1: Subfraction; RN2: Cinnamtannin B1; Hyalobarrier gel: Reference drug.

When the ovarian tissue sections were evaluated after immunohistochemical analysis, the percentages of TNF-α, IL-6, and IL-8 immunopositive areas (Figure 8, Table 7) were lower in the sham group samples compared to the other groups (TNF-α, IL-6, IL-8, respectively). However, this decrease was not statistically significant for IL-8. A statistically significant reduction in TNF-α immunoreactivity was observed in the RN2 group (16.30%) compared to the control group (21.36%; *p* = 0.029). Additionally, a notable decrease in IL-6 immunopositive areas was observed in the FR5 group (16.81%) compared to the control group (20.54%; *p* = 0.050). No statistically significant differences were found among the other groups.

**Table 7:** Ovarian Immunopositive Area Percentages^a^

Groups	Fibrotic area (%)	TNF-α immunopositive area percentage (%)	IL-6 immunopositive area percentage (%)	IL-8 immunopositive area percentage (%)
Sham	1.55 ± 0.16	1.58 ± 0.19	4.16 ± 0.68	1.93 ± 0.29
Control	2.85 ± 0.34	9.43 ± 0.62	16.17 ± 0.69	1.74 ± 0.22
Main extract	2.83 ± 0.40	7.18 ± 0.80	13.76 ± 0.67	2.39 ± 0.30
Aqueous subextract	3.10 ± 0.44	7.94 ± 0.56	13.70 ± 0.66	1.99 ± 0.22
FR5	2.90 ± 0.34	6.72 ± 0.44	12.78 ± 0.67*	2.26 ± 0.25
VLC1	3.04 ± 0.33	8.43 ± 0.53	13.81 ± 0.70	2.38 ± 0.33
RN2	3.10 ± 0.42	6.13 ± 0.39*	13.08 ± 0.60	2.68 ± 0.41
Hyalobarrier gel	2.50 ± 0.41	7.84 ± 0.89	14.20 ± 0.73	1.97 ± 0.20

a *p* < 0.05 (results were compared with the control group); Main extract: *Rumex nepalensis* extract; FR5: Fraction 5; VLC1: Subfraction; RN2: Cinnamtannin B1; Hyalobarrier gel: Reference drug.

**Figure 8:**
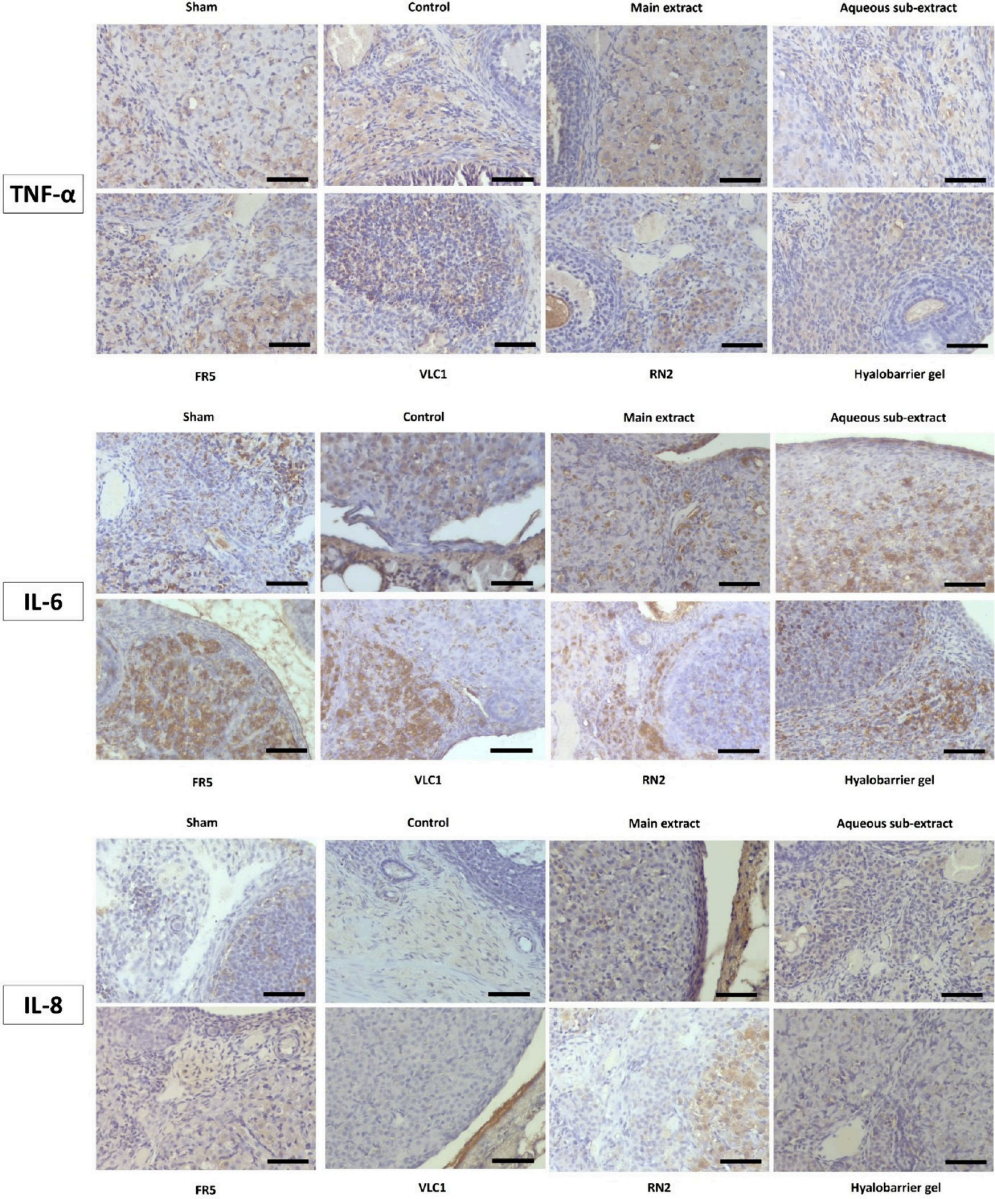
TNF-α: TNF-α immunoreactivity in ovarian sections of experimental groups (DAB, Mayer’s hematoxylin, X400). IL-6: IL-6 immunoreactivity in ovarian sections of experimental groups (DAB, Mayer’s hematoxylin, X400 scale bar 50 μm). IL-8: IL-8 immunoreactivity in ovarian sections of experimental groups (DAB, Mayer’s hematoxylin, X400); Main extract: *Rumex nepalensis* extract; FR5: Fraction 5; VLC1: Subfraction; RN2: Cinnamtannin B1; Hyalobarrier gel: Reference drug.

## 4 Discussion

Abdominal surgery is among the most frequently performed procedures, involving a range of organs and structures such as the esophagus, stomach, liver, pancreas, and kidneys. A significant complication arising from these surgeries is the development of postoperative adhesions within the peritoneal cavity. The adhesions can lead to several complications, although they are often asymptomatic. However, they can also result in serious conditions like small bowel obstruction, fistula formation, chronic abdominopelvic pain, infertility, ureteral obstruction, and postoperative bleeding. Studies have indicated that 33% of patients who have undergone previous surgeries require retreatment due to adhesion-related complications, and, in some cases, these complications can lead to mortality.^23,24^ Consequently, the increasing treatment costs associated with adhesions place a substantial economic burden on healthcare systems. As a result, extensive research has been directed toward preventing and managing adhesion formation. *In vitro* and *in vivo* studies aimed at discovering and developing new therapeutic agents have gained significant attention.

Previous preclinical studies have shown that several plant species, including *Rheum rhaponticum* L., *Rheum rhabarbarum* L., *Rubia cordifolia* L., *Rumex acetosa* L., *Asphodeline damascena* subsp. *rugosa* Tuzlacı, *A. tenuior* subsp. *tenuiflora* (K.Koch) Tuzlacı, and *A. cilicica* Tuzlacı, possess anticoagulant, antiplatelet, antioxidant, profibrinolytic, antithrombotic, fibrinolytic, and anti-inflammatory effects.^25–30^ Furthermore, compounds such as flavonoids, anthraquinones, and tannins have been reported to exhibit these beneficial activities.^17,31–36^ Building on this, we investigated the activities of *Asphodeline lutea* Rchb. (Asphodelaceae), *Rheum ribes* L. (Polygonaceae), *Rubia tinctorum* L. (Rubiaceae), and *Rumex nepalensis* Spreng. (Polygonaceae), which share similar group of compounds (flavonoids, anthraquinones, and tannins), to assess their potential to prevent adhesion formation. While the mentioned plants share these phytochemical features, each species also exhibits unique characteristics: *R. ribes* contains various anthraquinones and phenolics and is traditionally used for gastrointestinal disorders and wound healing;^37,38^ *R. tinctorum* is rich in alizarin and purpurin with antioxidant and enzyme-modulating activities;^39,40^ *A. lutea* roots are phenolic-rich with notable antioxidant capacity supporting wound healing and anti-inflammatory uses;^41^ and *R. nepalensis* has tannin-rich extracts contributing to antimicrobial and anti-inflammatory effects.^42,43^ Considering both their shared and distinguishing features, these species were selected to provide a comparative framework for evaluating their antiadhesive potential. Among these plant extract, *R. nepalensis* exhibited the highest fibrinolytic activity. Therefore, this plant was selected for the further activity studies. Adhesion formation resulting from fibrin accumulation after abdominal surgery shares similarities with the disrupted and nonlinear healing process observed in skin wounds. From a histopathological standpoint, the tissue repair mechanism involves several key stages, including hemostasis, inflammation, and regeneration. The findings of our study, demonstrating the antiadhesion effects of *R. nepalensis*, further support its traditional use in wound healing, as indicated by ethnobotanical records.^44,45^

*In vitro* activity-guided fractionation and isolation led to the identification of RN2 (cinnamtannin B1) and RN3 (epicatechin gallate) as the active compounds of *R. nepalensis*. RN2 exhibited a higher fibrinolytic activity (1.9911 U/mL plasmin equivalent) compared to epicatechin gallate (1.3371 U/mL plasmin equivalent). The extract, subextract, fraction, subfraction, and compound of *R. nepalensis*, exhibiting the highest fibrinolytic activity, were subjected to *in vivo* experiments, and their effects were comparatively evaluated. At the end of the experiment, the activities were assessed based on macroscopic observations, histopathological, immunohistochemical, and biochemical parameters. Notably, in the *in vivo* experiments, the bioactivity of the VLC1 subfraction, containing both RN2 and RN3, was prominent. This effect was hypothesized to be due to the enhanced effect of these two compounds when used together. Furthermore, FR5 and RN2 showed results comparable to the reference Hyalobarrier gel.

Pro-inflammatory cytokines (TNF-α, IL-1β, IL-2, IL-6, IL-8, IFN (Interferon)-γ) produced by lymphocytes or monocytes are known to be important in inflammation.^46^ Therefore, immunohistochemical evaluation of the mentioned pro-inflammatory cytokines was conducted as part of our study. When the percentages of TNF-α, IL-6 and IL-8 immunopositive areas in the uterine tissue were evaluated, an anti-inflammatory effect was generally observed in the treatment groups. The subfraction group VLC1 and RN2 were found to significantly reduce inflammatory cytokines statistically. Both *in vivo* and *in vitro* studies have previously indicated that polyphenols regulate multiple inflammatory responses by modulating cytokine activity via macrophages.^47^ Various phenolic compounds have been shown to inhibit pro-inflammatory cytokine and chemokine expression in LPS-activated mouse macrophages, human mast cells, astrocytes, synovial cells, and peripheral blood mononuclear cells.^48–52^

Moreover, polyphenolic compounds have been reported to exhibit anti-inflammatory activity by reducing the production of IL-6 and TNF-α due to their immunomodulatory effects.^53,54^ This study also demonstrated that the root extract of *R. nepalensis* exhibits anti-inflammatory effects by decreasing TNF-α, IL-6, and IL-8 levels compared to the control group, likely mediated by its polyphenolic compounds.

Interestingly, previous research has shown that cinnamtannin B1, the main bioactive compound found in *Cinnamomum validinerve*, can reduce immune cell infiltration and lower pro-inflammatory cytokine levels, including TNF-α and IL-6, when administered intraperitoneally in infection models.^55^ Similarly, in rat models of hepatic fibrosis induced by carbon tetrachloride, compounds like epicatechin gallate and epigallocatechin gallate, derived from *Camellia sinensis*, effectively suppressed key inflammatory markers such as IL-17, transforming growth factor-beta (TGF-β), and TNF-α. These natural compounds also alleviated oxidative stress and significantly improved liver histology, highlighting their potential in antifibrotic therapies.^56^ Our findings are consistent with those reported previously.

In histopathological and immunohistochemical analyses, the percentage of fibrotic areas in the VLC1 and RN2 groups was found to be significantly lower compared to the control group. When evaluating the immunopositive areas for TNF-α, IL-6, and IL-8 in the uterine tissues of the experimental groups, a significant decrease in TNF-α immunoreactivity was observed in the RN2 group compared to the control, extract, and subextract groups. The percentage of IL-6 immunopositive areas in the primary extract and VLC1 groups was significantly lower than in the control group, while the lowest IL-8 immunoreactivity percentage among the treatment groups was found in the RN2 group. No statistically significant difference was observed when comparing the percentages of fibrotic areas in ovarian sections between the experimental groups. Upon evaluating ovarian tissue sections after immunohistochemical analysis, a statistically significant decrease in TNF-α immunoreactivity was observed in the RN2 group compared to the control group. A significant decrease in the percentage of IL-6 immunopositive areas was also found in the FR5 group compared to the control group. In conclusion, based on the histopathological and immunohistochemical analysis findings, a notable improvement was observed in the VLC1 and RN2 groups, and this result was consistent with the macroscopic analysis findings.

Plasmin, which is the active form of plasminogen plays a key role in fibrinolysis by breaking down fibrin-based adhesion tissue. It is activated by urokinase-type plasminogen activator and tissue-type plasminogen activator (tPA), and inhibited by plasminogen activator inhibitor-1 (PAI-1). PAI-1, produced and secreted by macrophages, platelets, endothelial cells, mesothelial cells, and fibroblasts, is regulated by various stimuli including thrombin, endotoxins, TGF-β, IL-1, TNF, and macrophage activity. Studies have shown that in patients with intra-abdominal adhesions, increased levels of PAI-1 and decreased levels of tPA in the peritoneal tissue lead to reduced fibrinolytic activity, consequently leading to the formation of postoperative adhesions.^57^ In studies conducted on plant extracts carrying tannin group compounds, it has been shown that *Potentilla erecta* (L.) Raeusch rhizome extract with high procyanidin and ellagitannin content causes an increase in tPA activity.^58^ Corilagin, an ellagitannin compound isolated from the aerial parts of *Phyllanthus urinaria* L. in the methanol extract, has been reported to enhance tPA activity and significantly reduce PAI activity in rat plasma.^59^ It has been reported that the ethanol extract of *Camellia sinensis*, rich in catechin derivatives, prevents adhesion formation when applied to the abdominal cavity of Wistar albino rats.^60^ In summary, tannin-derived compounds, as well as the plants containing these compounds, have demonstrated beneficial effects against inflammation, collagen accumulation, and oxidative stress.

Peritoneal injury triggers a cascade of biochemical process, including the activation of the coagulation pathway accompanied by the release of numerous mediators. The peritoneal surface and its remesothelialization within 5–8 days play a crucial role in adhesion formation. The pathophysiology of adhesions involves mesothelial cells, macrophages, neutrophils, leukocytes, and fibrin as primary factors. Following surgical trauma, the increased number of macrophages enhances activity within the peritoneal cavity and promotes the production of various cytokines and enzymes, including IL-1, IL-6, TNF, leukotriene B4, prostaglandin E2 (PGE2), cyclooxygenase (COX) and lipoxygenase (LOX) metabolites, plasminogen activator, PAI, collagenase, and elastase.^61–63^ Mesothelial cells located at the injury site contribute significantly to the onset of serosal inflammation, fibrosis, and the healing process by secreting a range of mediators. These mediators include growth factors such as TGF-β, EGF, vascular endothelial growth factor (VEGF), IGF, and FGF, alongside cytokines, chemokines, coagulation cascade components, prostaglandins, prostacyclins, reactive nitrogen and oxygen species, antioxidant enzymes, and extracellular matrix proteins.^64,65^

Activity studies conducted on cinnamtannin B1 have demonstrated its antioxidant and anticancer properties. It has been reported that cinnamtannin B1 exhibits strong antiproliferative activity against HepG2 and SiHa cell lines, and its anticancer mechanism is associated with cell cycle arrest and the induction of apoptosis. Its ability to induce apoptosis selectively in tumor cells, while exerting antiapoptotic effects in normal cells, has been proposed as a potential basis for the development of targeted therapeutic strategies.^66,67^ In another study, cinnamtannin B1 was found to promote wound healing by stimulating *in vivo* mesenchymal stem cell migration.^68^

In the peritoneal cavity, tissue plasminogen activator (tPA), TGF-β1, tumor necrosis factor-alpha (TNF-α), interleukin-6 (IL-6), matrix metalloproteinases (MMPs), VEGF, cyclooxygenases (COX), and oxidative stress-related factors have been reported to function as key regulatory elements in the biochemical cascades involved in adhesion formation.^69^ MMPs are proteolytic enzymes in adhesion tissue and the peritoneum that contribute to remodeling the extracellular matrix (ECM) and play a crucial role in the normal healing process. TGF-β1 has been shown to stimulate MMP activity. When peritoneal fibroblasts were exposed to TGF-β1, an increase in MMP-2 and MMP-9 mRNA expression was observed.^70^ MMPs are responsible for the degradation of ECM proteins, and their proteolytic activity is regulated by tissue inhibitors of metalloproteinases (TIMPs). In hypoxia, increased TIMP release causes ECM formation, shaping and increased tissue fibrosis.^71,72^ It has been demonstrated that catechins regulate ECM formation through the regulation of MMP-2 activity, thereby contributing to the wound healing process by preventing the excessive accumulation of fibrotic tissue.^56,73^ It has been suggested that the tannin-derivatives may have inhibited fibrin tissue accumulation through the regulation of MMP activity.

## 5 STUDY LIMITATION

The present study has some limitations. The two bioactive compounds obtained in limited amounts and in partially purified form. Further purification or chromatographic confirmation could not be performed due to the restricted quantity of material and resource constraints. In designing this study, we followed the 3Rs principle to minimize animal use; however, it should be noted that the *in vitro* assays cannot fully replicate the complex *in vivo* environment. Additionally, formal synergy assessments between cinnamtannin B1 and epicatechin gallate were not performed. The study duration was relatively short, and only a single-sex animal group was used, while toxicity data were not collected. These limitations should be considered when interpreting the findings and will be addressed in future investigations.

## 6 CONCLUSION

This study demonstrates for the first time that *in vitro* fibrinolytic activity measured using the fibrin plate method can effectively guide the selection of plant extracts for *in vivo* testing in a rat model of intra-abdominal adhesions. Using this approach, ineffective samples can be eliminated, and those with high fibrinolytic potential, can be prioritized, reducing the need for extensive animal experimentation. The results of the present study highlight the promise of tannin-rich plant extracts and their bioactive constituents as effective agents for preventing intra-abdominal adhesions. A subfraction containing cinnamtannin B1 and (−)-epicatechin gallate significantly reduced adhesion formation *in vivo*, suggesting that the two compounds exert complementary effects. Therefore, the underlying mechanism and the optimal combination ratios remain to be clarified. Future studies should therefore aim to (i) conduct comprehensive phytochemical profiling, (ii) perform large-scale isolation and improved purification, (iii) include quantitative kinetic assays. (iv) clarify the molecular mechanisms and possible synergistic interactions, (v) establish optimal dosages, formulations, and delivery methods, (vi) evaluate pharmacokinetics, safety, and bioavailability, (vi) conduct long-term toxicological assessments. Together, these steps will provide a stronger foundation for the preclinical development and eventual clinical translation of these compounds.

## Supplementary Material



## Notes

Spectral data and NMR data table for isolated compounds (PDF)

## Abbreviations

1D and 2D NMR: one- and two-dimensional nuclear magnetic resonance spectroscopy
AlE: *Asphodeline lutea* extract
ANOVA: analysis of variance
ARRIVE: Animal Research: Reporting of *in vivo* Experiments
CHCl_3_: chloroform
COSY: correlation spectroscopy
COX: cyclooxygenase
DAB: diaminobenzidine
DCM: dichloromethane
ECM: extracellular matrix
FR: fraction
G.Ü.ET: Experimental Animal Ethics Committee of Gazi University
H&E: hematoxylin and eosin
HMBC: heteronuclear multiple bond correlation
HSQC: heteronuclear single quantum coherence
HR-MS: high resolution mass spectrometry
IFN: interferon
IL: interleukin
LOX: lipoxygenase
MeOH: methanol
MMP: matrix metalloprotease
MS: mass spectrometry
NMR: nuclear magnetic resonance
PAA: postoperative abdominal adhesion
PAI: plasminogen activator inhibitor
PBS: phosphate-buffered saline
PGE: prostaglandin E2
Prep TLC: preparative thin layer chromatography
RnE: *Rumex nepalensis* extract
RN2: cinnamtannin B1
RN3: (−)-epicatechin gallate
RrE: *Rheum ribes* extract
RtE: *Rubia tinctorum* extract
SEM: standard error of the mean
TIMPs: tissue inhibitors of metalloproteinases
TLC: thin-layer chromatography
TNF: tumor necrosis factor
tPA: tissue plasminogen activator
U: unit
UV: ultraviolet
VEGF: vascular endothelial growth factor
VLC: vacuum liquid chromatography

## References

[1] Brüggmann D., Tchartchian G., Wallwiener M., Münstedt K., Tinneberg H. R., Hackethal A. (2010). Intra-abdominal adhesions: definition,
origin, significance in surgical practice, and treatment options. Dtsch. Arztebl. Int..

[2] Arung W., Meurisse M., Detry O. (2011). Pathophysiology
and prevention of
postoperative peritoneal adhesions. World J.
Gastroenterol..

[3] Ten
Broek R. P. G., Krielen P., Di Saverio S., Coccolini F., Biffl W. L., Ansaloni L., Velmahos G. C., Sartelli M., Fraga G. P., Kelly M. D., Moore F. A., Peitzman A. B., Leppaniemi A., Moore E. E., Jeekel J., Kluger Y., Sugrue M., Balogh Z. J., Bendinelli C., Civil I., Coimbra R., De Moya M., Ferrada P., Inaba K., Ivatury R., Latifi R., Kashuk J. L., Kirkpatrick A. W., Maier R., Rizoli S., Sakakushev B., Scalea T., Søreide K., Weber D., Wani I., Abu-Zidan F. M., De’Angelis N., Piscioneri F., Galante J. M., Catena F., van Goor H. (2018). Bologna guidelines
for diagnosis and management of adhesive small bowel obstruction (ASBO):
2017 update of the evidence-based guidelines from the world society
of emergency surgery ASBO working group. World
J. Emerg Surg..

[4] Awonuga A. O., Fletcher N. M., Saed G. M., Diamond M. P. (2011). Postoperative adhesion
development following cesarean and open intra-abdominal gynecological
operations. Reprod. Sci..

[5] Sikirica V., Bapat B., Candrilli S. D., Davis K. L., Wilson M., Johns A. (2011). The inpatient burden
of abdominal and gynecological adhesiolysis
in the US. BMC Surgery..

[6] Davis C. C., Choisy P. (2024). Medicinal plants meet
modern biodiversity science. Curr. Biol..

[7] Tareq A. M., Farhad S., Neshar Uddin A. B. M., Hoque M., Nasrin M. S., Uddin M. M. R., Hasan M., Sultana A., Munira M. S., Lyzu C., Moazzem Hossen S. M., Ali Reza A. S. M., Emran T. B. (2020). Chemical
profiles, pharmacological properties, and in silico studies provide
new insights on *Cycas pectinata*. Heliyon..

[8] Nur
Manik M. I., Ali M. H., Islam M. M., Zobayed A., Saadullah S., Khan A., Tabassum F., Noor F. (2022). In vitro antioxidant,
cytotoxic, thrombolytic activities and phytochemical evaluation of
methanol extract of the *Ampelocissus barbata* (wall.)
leaves. Biomed Pharmacol J..

[9] Rahman M., Surag A. T., Begum R., Hossain Tusher M. S., Huda M. K. (2022). Phytochemical, antioxidant, anti-ınflammatory,
and thrombolytic properties of *Cleisomeria lanatum* (Lindl.) Lindl. ex G. Don. Scientifica.

[10] Astrup T., Mullertz S. (1952). The fibrin plate method
for estimating fibrinolytic
activity. Arch. Biochem. Biophys..

[11] Nguyen T. M. H., Nguyen T. T. O., Le N. T., Spyridovich E. V., Nguyen V. H., Chau V. M. (2021). Preliminary observation
on the fibrinolytic
activity of *Dimocarpus longan* seed. Chem. Nat. Compd..

[12] Süntar I., Demirel M. A., Ceribasi A. O., Ergin I., Gökbulut A. (2021). Preventive
effect of Rumex crispus L. on surgically induced intra-abdominal adhesion
model in rats. DARU J. Pharm. Sci..

[13] Allègre L., Le Teuff I., Leprince S., Warembourg S., Taillades H., Garric X., Letouzey V., Huberlant S. (2018). A new bioabsorbable
polymer film to prevent peritoneal adhesions validated in a post-surgical
animal model. PLoS One..

[14] Sahbaz A., Aynioglu O., Isik H., Gulle K., Akpolat
Ferah M., Cicekler Sahbaz H. (2015). Cholecalciferol (Vitamin D3) prevents
postoperative adhesion formation by inactivating the nuclear factor
kappa B pathway: A randomized experimental study. J. Surg. Res..

[15] Türkoğlu A. M., Gül H. K., Yuksel, Alabalik U., Ülger B. V., Uslukaya O., Avci Y. (2015). Effect of intraperitoneal
curcumin
instillation on postoperative peritoneal adhesions. Med. Princ. Pract..

[16] Wang C., Li X., Meng X., Zhou J., Qin F., Hou L. (2014). Prevention
of experimental postoperative peritoneal adhesions through the intraperitoneal
administration of tanshinone IIA. Planta Med..

[17] Zhang H., Song Y., Li Z., Zhang T., Zeng L. (2016). Evaluation
of breviscapine on prevention of experimentally induced abdominal
adhesions in rats. Am. J. Surg..

[18] Celepli S., Kısmet K., Kaptanoğlu B., Erel S., Ozer S., Celeplı P., Kaygusuz G., Devrım E., Gencay O., Sorkun K., Durak I., Akkuş M. A. (2011). The effect
of oral honey and pollen on postoperative intra-abdominal adhesions. Turk. J. Gastroenterol..

[19] Saribas G. S., Ozogul C., Tiryaki M., Alpaslan Pinarli F., Hamdemir Kilic S. (2020). Effects of uterus derived mesenchymal
stem cells and
their exosomes on Asherman’s syndrome. Acta Histochem..

[20] Kamiya K., Watanabe C., Endang H., Umar M., Satake T. (2001). Studies on
the constituents of bark of *Parameria laevigata* Moldenke. Chem. Pharm. Bull..

[21] Özgünseven, A. *Potentilla speciosa* Willd. var. speciosa Willd. ve sekonder metabolitlerinin *α*-glukozidaz ve tirosinaz inhibitör etkilerinin araştırılması. Yüksek Lisans Tezi, Hacettepe Üniversitesi Sağlık Bilimleri Enstitüsü, Ankara, 2021, 109–113.

[22] Davis A. L., Cai Y., Davies A. P., Lewis J. R. (1996). 1H and 13C NMR assignments of some
green tea polyphenols. Magn. Reson. Chem..

[23] Kocaay A. F., Çelik S. U., Eker T., Çetinkaya Ö.A., Genç V. (2015). İntraperitoneal
adezyonlar: Patogenezi, klinik
önemi ve önleme stratejileri. Med. Bull. Sisli Etfal Hosp..

[24] Menzies D. (1993). Postoperative
adhesions: Their treatment and relevance in clinical practice. Ann. R. Coll. Surg. Engl..

[25] Chen Y., Chen P. D., Bao B. H., Shan M. Q., Zhang K. C., Cheng F. F., Cao Y. D., Zhang L., Ding A. W. (2018). Anti-thrombotic
and pro-angiogenic effects of *Rubia cordifolia* extract
in zebrafish. J. Ethnopharmacol..

[26] Zengin G., Locatelli M., Ferrante C., Menghini L., Orlando G., Brunetti L., Recinella L., Chiavaroli A., Leone S., Leporini L., Aumeeruddy M. Z., Mahomoodally M. F. (2019). New pharmacological targets of three *Asphodeline* species using in vitro and ex vivo models of
inflammation and oxidative
stress. Int. J. Health Res..

[27] Jeong D., Irfan M., Lee D. H., Hong S. B., Oh J. W., Rhee H. (2020). *Rumex acetosa* modulates platelet function and inhibits
thrombus formation in rats. BMC Complement.
Med. Ther..

[28] Wang K., Gao L., Zhang Q., Yao W., Zhang M., Tang Y., Ding A., Zhang L. (2020). Revealing the mechanisms and the
material basis of Rubia cordifolia L. on abnormal uterine bleeding
with uniting simultaneous determination of four components and systematic
pharmacology approach-experimental validation. J. Pharm. Biomed. Anal..

[29] Jeon B. R., Irfan M., Lee S. E., Lee J. H., Rhee M. H. (2022). *Rumex acetosella* inhibits platelet function via impaired
MAPK and phosphoinositide 3-kinase signaling. Chin. J. Integr. Med..

[30] Liudvytska O., Ponczek M. B., Krzyżanowska-Kowalczyk J., Kowalczyk M., Balcerczyk A., Kolodziejczyk-Czepas J. (2023). Effects of *Rheum rhaponticum* and *Rheum rhabarbarum* extracts on haemostatic activity of blood plasma components and
endothelial cells in vitro. J. Ethnopharmacol..

[31] Wei G., Wu Y., Gao Q., Zhou C., Wang K., Shen C., Wang G., Wang K., Sun X., Li X. (2017). Effect of
emodin on preventing postoperative intra-abdominal adhesion formation. Oxid. Med. Cell. Longev..

[32] Wei G., Wu Y., Gao Q., Shen C., Chen Z., Wang K., Yu J., Li X., Sun X. (2018). Gallic acid attenuates postoperative
intraabdominal adhesion by inhibiting inflammatory reaction in a rat
model. Med. Sci. Monit..

[33] Javanmardi S., Golmohammadi S., Mazaheri-Khamene R. (2017). Evaluation of silymarin effects on
post-operative peritoneal adhesion in rats. J. Urmia Univ. Med. Sci..

[34] Jedinák A., Maliar T., Grancai D., Nagy M. (2006). Inhibition activities
of natural products on serine proteases. Phytother.
Res..

[35] Mozzicafreddo M., Cuccioloni M., Eleuteri A. M., Fioretti E., Angeletti M. (2006). Flavonoids
inhibit the amidolytic activity of human thrombin. Biochimie..

[36] Mozzicafreddo M., Cuccioloni M., Bonfili L., Eleuteri A. M., Fioretti E., Angeletti M. (2008). Antiplasmin
activity of natural occurring polyphenols. Biochim.
Biophys. Acta.

[37] Naqishbandi A. M., JA¤ger A. K., Al-Khateeb E. H. (2009). A comparative qualitative and quantitative
study of Anthraquinone derivatives in the roots of *Rheum ribes* and *Rheum emodi* by HPLC. Iraqi J. Pharm. Sci..

[38] Keser S., Keser F., Karatepe M., Kaygili O., Tekin S., Turkoglu I., Demir E., Yilmaz O., Kirbag S., Sandal S. (2020). Bioactive contents, *In vitro* antiradical,
antimicrobial and cytotoxic properties of rhubarb (*Rheum ribes* L.) extracts. Nat. Prod Res..

[39] Langa-Lomba N., Sánchez-Hernández E., Buzón-Durán L., González-García V., Casanova-Gascón J., Martín-Gil J., Martín-Ramos P. (2021). Activity of anthracenediones and
flavoring phenols in hydromethanolic extracts of *Rubia tinctorum* against grapevine phytopathogenic fungi. Plants..

[40] Wang W., Zhang J., Qi W., Su R., He Z., Peng X. (2021). Alizarin and purpurin from *Rubia tinctorum* L. suppress
ınsulin fibrillation and reduce the amyloid-ınduced cytotoxicity. ACS Chem. Neurosci..

[41] Lazarova I., Zengin G., Aktumsek A., Gevrenova R., Ceylan R., Uysal S. (2014). HPLC-DAD analysis of
phenolic compounds
and antioxidant properties of *Asphodeline lutea* roots
from Bulgaria and Turkey. Industrial Crops and
Products..

[42] Cao Y. J., Pu Z. J., Tang Y. P., Juan S., Chen Y. Y., Kang A., Guisheng Z., Duan J. A. (2017). Advances in bio-active
constituents, pharmacology and clinical applications of rhubarb. Chin Med..

[43] Chelly M., Chelly S., Occhiuto C., Cimino F., Cristani M., Saija A., Muscarà C., Ruberto G., Speciale A., Bouaziz-Ketata H., Siracusa L. (2021). Comparison of phytochemical profile
and bioproperties of methanolic extracts from different parts of tunisian *Rumex roseus*. Chem. Biodivers..

[44] Pradhan B. K., Badola H. K. (2008). Ethnomedicinal plant use by Lepcha tribe of Dzongu
Valley, bordering Khangchendzonga Biosphere Reserve, in North Sikkim, *India*. J. Ethnobiol. Ethnomed..

[45] Kumar S., Joseph L., George M., Kaur L., Bharti V. (2011). Skeletal muscle
relaxant activity of methanolic extract of *Rumex nepalensis* in albino rats. J. Chem. Pharm. Res..

[46] González R., Ballester I., López-Posadas R., Suárez M. D., Zarzuelo A., Martínez-Augustin O., Sánchez
de Medina F. (2011). Effects of flavonoids and other polyphenols on inflammation. Crit. Rev. Food Sci. Nutr..

[47] Comalada M., Ballester I., Bailón E., Sierra S., Xaus J., Gálvez J., de Medina F. S., Zarzuelo A. (2006). Inhibition of proinflammatory
markers in primary bone marrow-derived mouse macrophages by naturally
occurring flavonoids: analysis of the structure-activity relationship. Biochem. Pharmacol..

[48] Blonska M., Czuba Z. P., Krol W. (2003). Effect of
flavone derivatives on
interleukin-1β (IL-1β) mRNA expression and IL-1β
protein synthesis in stimulated RAW 264.7 macrophages. Scand. J. Immunol..

[49] Lyu S. Y., Park W. B. (2005). Production of cytokine
and NO by RAW 264.7 macrophages
and PBMC in vitro incubation with flavonoids. Arch. Pharm. Res..

[50] Min Y. D., Choi C. H., Bark H., Son H. Y., Park H. H., Lee S., Park J. W., Park E. K., Shin H. I., Kim S. H. (2007). Quercetin
inhibits expression of inflammatory cytokines through attenuation
of NF-κB and p38 MAPK in HMC-1 human mast cell line. Inflamm. Res..

[51] Sharma V., Mishra M., Ghosh S., Tewari R., Basu A., Seth P., Sen E. (2007). Modulation
of interleukin-1β
mediated inflammatory response in human astrocytes by flavonoids:
implications in neuroprotection. Brain Res.
Bull..

[52] Drummond E. M., Harbourne N., Marete E., Martyn D., Jacquier J., O’Riordan D., Gibney E. R. (2013). Inhibition of proinflammatory biomarkers
in THP1 macrophages by polyphenols derived from chamomile, meadowsweet
and willow bark. Phytother. Res..

[53] Schindler R., Mancilla J., Endres S., Ghorbani R., Clark S. C., Dinarello C. A. (1990). Correlations
and interactions in the production of
interleukin-6 (IL-6), IL-1, and tumor necrosis factor (TNF) in human
blood mononuclear cells: IL-6 suppresses IL-1 and TNF. Blood..

[54] Lamy S., Akla N., Ouanouki A., Lord-Dufour S., Béliveau R. (2012). Diet-derived polyphenols inhibit
angiogenesis by modulating
the interleukin-6/STAT3 pathway. Exp. Cell Res..

[55] Yang C. L., Wu H. C., Hwang T. L., Lin C. H., Cheng Y. H., Wang C. C., Kan H. L., Kuo Y. H., Chen I. S., Chang H. S., Lin Y. C. (2020). Anti-inflammatory and antibacterial
activity constituents from the stem of *Cinnamomum validinerve*. Molecules.

[56] Wang L., Yang G., Yuan L., Yang Y., Zhao H., Ho C. T., Li S. (2019). Green tea
catechins effectively altered
hepatic fibrogenesis in rats by inhibiting ERK and Smad1/2 phosphorylation. J. Agric. Food Chem..

[57] Deynez G., Miser Salihoğlu E., Süntar İ. (2023). The
role of anticoagulant, thrombolytic, and fibrinolytic activities in
the prevention of peritoneal adhesion. Trakya
Univ. J. Nat. Sci..

[58] Marcinczyk N., Gołaszewska A., Gromotowicz-Popławska A., Misztal T., Strawa J., Tomczyk M., Kasacka I., Chabielska E. (2021). Multidirectional effects of tormentil extract on hemostasis
in experimental diabetes. Front. Pharmacol..

[59] Shen Z.-Q., Dong Z.-J., Peng H., Liu J.-K. (2003). Modulation of PAI-1
and tPA activity and thrombolytic effects of corilagin. Planta Med..

[60] Parsaei P., Karimi M., Asadi S. Y., Rafieian-Kopaei M. (2013). Bioactive
components and preventive effect of green tea (*Camellia sinensis*) extract on post-laparotomy intra-abdominal adhesion in rats. Int. J. Surg..

[61] DiZerega, G. S. The peritoneum: postsurgical repair and adhesion formation. In Female Reproductive Surgery; Rock, J. A. , Murphy, A. A. , Jones, H. W. , Eds.; Williams & Wilkins: Baltimore, MD, 1992; pp 2–18.

[62] Rodgers K. E., DiZerega G. S. (1993). Function of peritoneal
exudate cells after abdominal
surgery. J. Investig. Surg..

[63] DiZerega G. S. (1997). Biochemical
events in peritoneal tissue repair. Eur. J.
Surg..

[64] Boland G.
M., Weigel R. J. (2006). Formation
and prevention of postoperative abdominal
adhesions. J. Surg. Res..

[65] Mutsaers S. E., Whitaker D., Papadimitriou J. M. (2002). Stimulation
of mesothelial cell proliferation
by exudate macrophages enhances serosal wound healing in a murine
model. Am. J. Pathol..

[66] López J. J., Jardín I., Salido G. M., Rosado J. A. (2008). Cinnamtannin B-1
as an antioxidant and platelet aggregation inhibitor. Life Sci..

[67] Wen L., You L., Yang X., Yang J., Chen F., Jiang Y., Yang B. (2015). Identification
of phenolics in litchi and evaluation of anticancer
cell proliferation activity and intracellular antioxidant activity. Free Radic. Biol. Med..

[68] Fujita K., Kuge K., Ozawa N., Sahara S., Zaiki K., Nakaoji K., Hamada K., Takenaka Y., Tanahashi T., Tamai K., Kaneda Y., Maeda A. (2015). Cinnamtannin B-1 promotes
migration of mesenchymal stem cells and accelerates wound healing
in mice. PLoS One..

[69] Braun K. M., Diamond M. P. (2014). The biology of adhesion
formation in the peritoneal
cavity. Semin. Pediatr. Surg..

[70] Cheong Y. C., Laird S. M., Li T. C., Shelton J. B., Ledger W. L., Cooke I. D. (2001). Peritoneal healing
and adhesion formation/reformation. Hum. Reprod.
Update.

[71] Alpay Z., Saed G. M., Diamond M. P. (2006). Female infertility and free radicals:
potential role in adhesions and endometriosis. J. Soc. Gynecol. Investig..

[72] Saed G. M., Diamond M. P. (2004). Molecular characterization of postoperative
adhesions:
the adhesion phenotype. J. Am. Assoc. Gynecol.
Laparosc..

[73] Sartor L., Pezzato E., Dell’Aica I., Caniato R., Biggin S., Garbisa S. (2002). Inhibition of matrix-proteases
by polyphenols: chemical
insights for anti-inflammatory and anti-invasion drug design. Biochem. Pharmacol..

[74] Leach R., Burns J., Dawe E., Smith Barbour M., Diamond M. (1998). Reduction of postsurgical adhesion
formation in the
rabbit uterine horn model with use of hyaluronate/carboxymethylcellulose
gel. Fertil. Steril..

