# Supplementary material for: Fibrinolytic and Anti-Abdominal Adhesion Effects of Tannin Derivatives from Rumex nepalensis Spreng.: Activity-Guided Isolation and Evaluation in a Postoperative Rat Model
Source: ACS Omega. 2025 Dec 5;10(49):60532–46. doi: 10.1021/acsomega.5c08121 (PMC12713443; doi:10.1021/acsomega.5c08121)
Supplement: Supplementary file 1 [file ao5c08121_si_001.pdf]

Supporting Information

Fibrinolytic and Anti-Abdominal Adhesion Effects of Tannin Derivatives from Rumex nepalensis Spreng.: Activity-Guided Isolation and Evaluation in Postoperative Rat Model

Gizem Deynez[a,b], İpek Süntar[c,*], Mürşide Ayşe Demirel[d], Saadet Özen Akarca Dizakar[e], Vahap Murat Kutluay[f], Ece Salihoğlu[g], Ayşe Kuruüzüm Uz[f], Osman Tugay[h]

[a]General Directorate of Public Hospitals, Ministry of Health, 06800 Çankaya, Ankara, Türkiye
[b]Health Sciences Institute, Gazi University, 06540 Çankaya, Ankara, Türkiye
[c]Department of Pharmacognosy Faculty of Pharmacy, Gazi University, 06330 Yenimahalle, Ankara, Türkiye tel: 00903122023176; E-mail:ipesin@gazi.edu.tr
[d]Department of Basic Pharmaceutical Sciences, Faculty of Pharmacy, Gazi University, 06330 Yenimahalle, Ankara, Türkiye
[e]Department of Histology and Embryology, Faculty of Medicine, Bakırçay University, 35665 Menemen, İzmir, Türkiye
[f]Department of Pharmacognosy, Faculty of Pharmacy, Hacettepe University, 06100 Çankaya, Ankara, Türkiye
[g]Department of Biochemistry, Faculty of Pharmacy, Gazi University, 06330 Yenimahalle, Ankara, Türkiye
[h]Department of Pharmaceutical Botany, Faculty of Pharmacy, Selçuk University, 42130 Selçuklu, Konya, Türkiye

List of Supplementary Material

HR-ESI-MS, and NMR spectra of compounds

Cinnamtannin B1 (RN2)

Figure	Caption	Page
**Figure S1**	HR-ESI-MS Spectrum of Cinnamtannin B1 (RN2)	S2
**Figure S2, S3, S4**	^{1}H-NMR Spectrum of Cinnamtannin B1 (RN2)	S3-S5
**Figure S5, S6, S7**	^{13}C-NMR spectrum of Cinnamtannin B1 (RN2)	S6-S8
**Figure S8**	COSY spectrum of Cinnamtannin B1 (RN2)	S9
**Figure S9**	HSQC spectrum of the Cinnamtannin B1 (RN2)	S10
**Figure S10**	HMBC spectrum of the Cinnamtannin B1 (RN2)	S11
**Table S1**	Assignments of ^{1}H NMR and ^{13C} NMR signals for Cinnamtannin B1 (RN2)	S12-S13

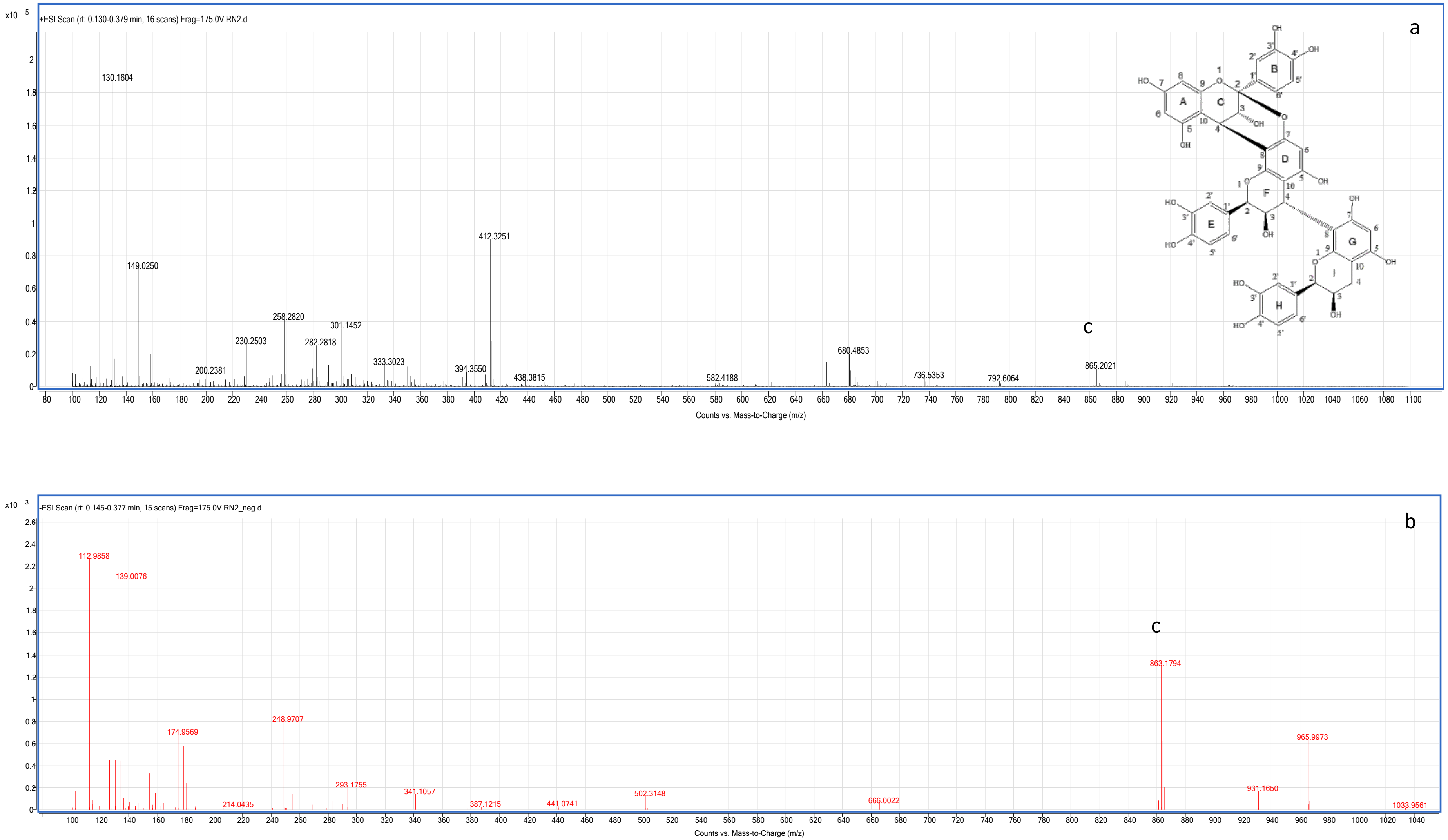

Figure S1. HR-ESI-MS Spectrum of Cinnamtannin B1 (RN2): a) +ESI Mass Spectrum b) -ESI Mass Spectrum c) Molecular Ion Peak

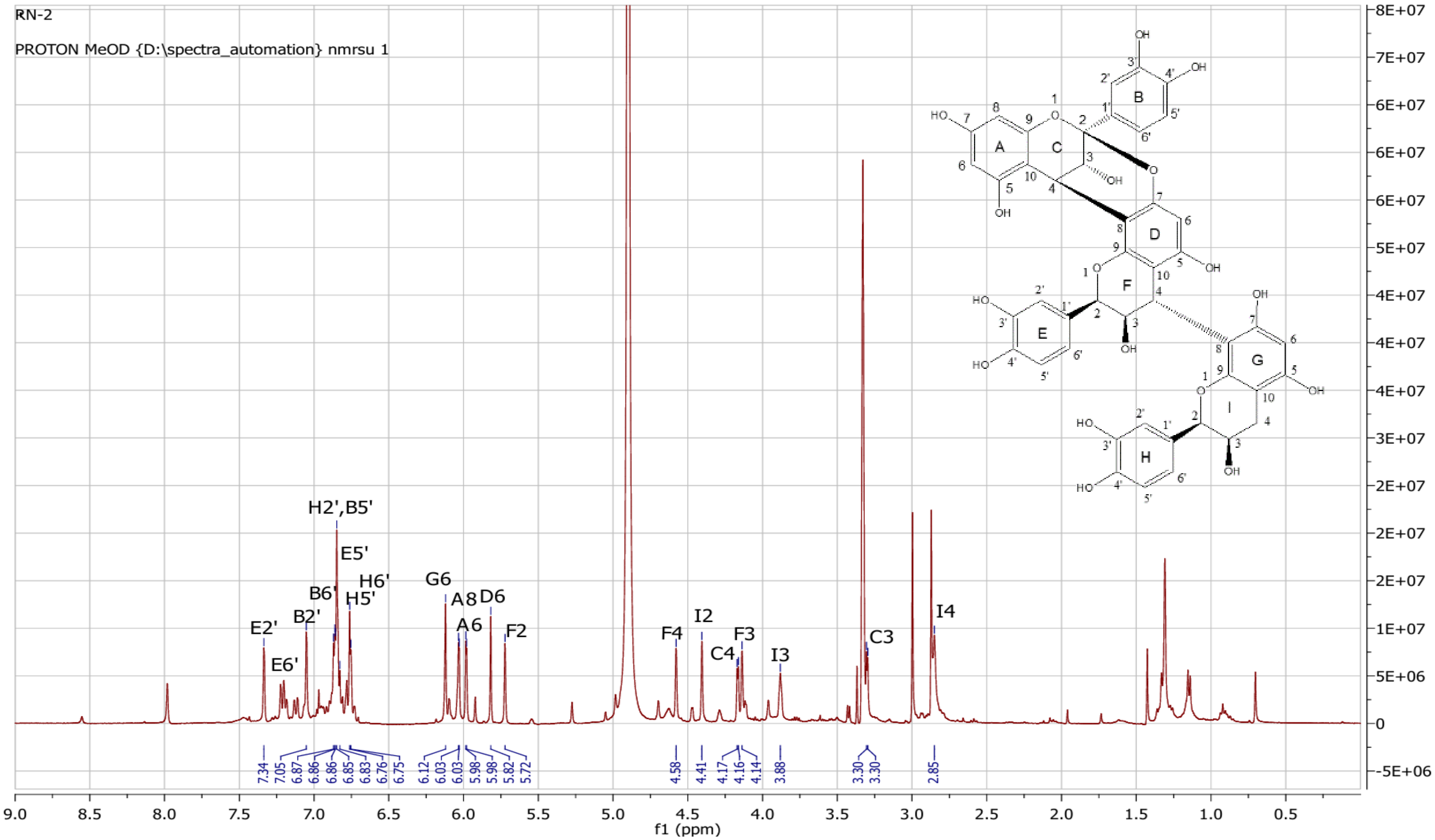

Figure S2. ^{1}H-NMR Spectrum of Cinnamtannin B1 (RN2) (CD_3OD, 400 MHz)

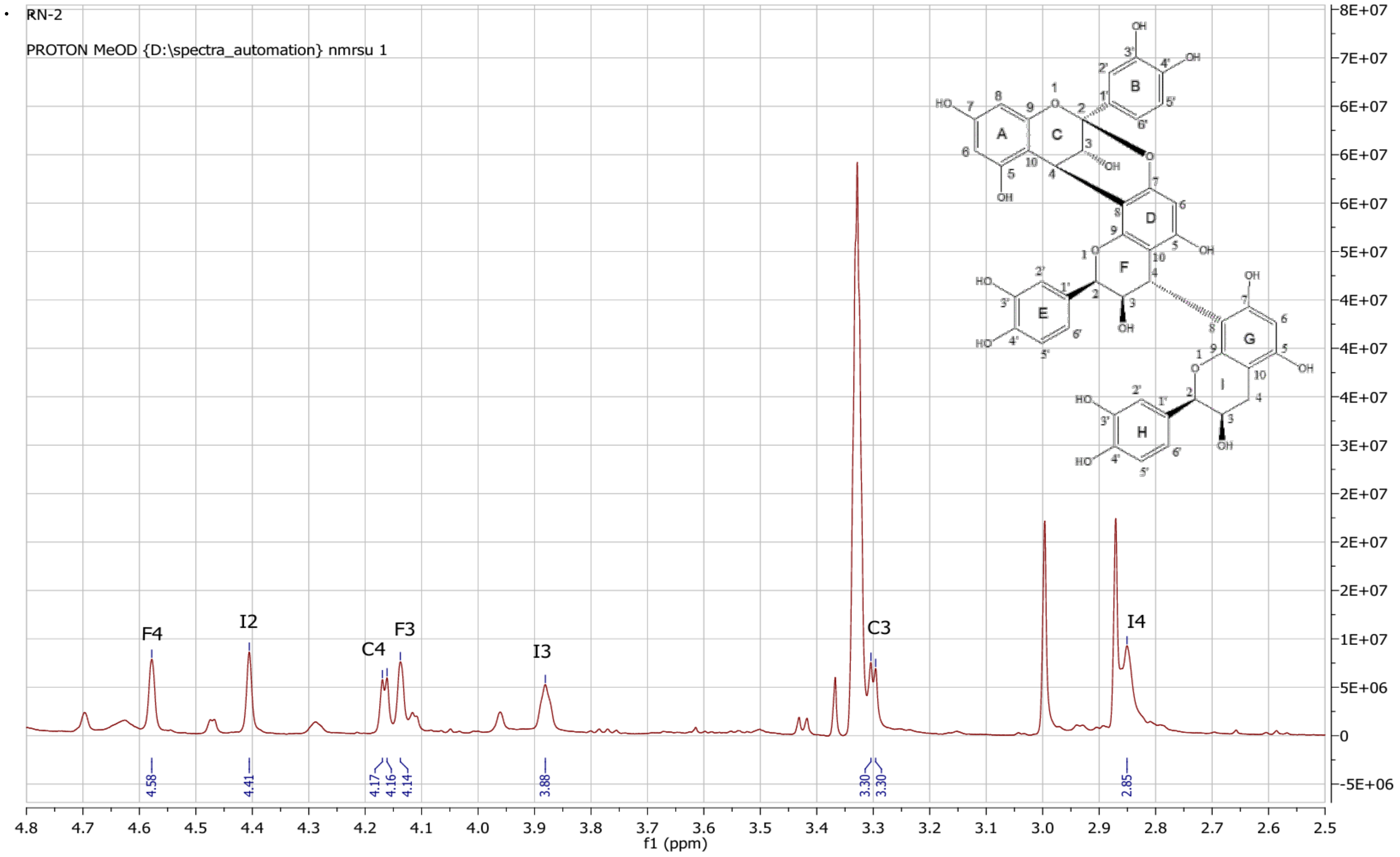

Figure S3. ^{1}H-NMR Spectrum of Cinnamtannin B1 (RN2) (CD_3OD, 400 MHz)

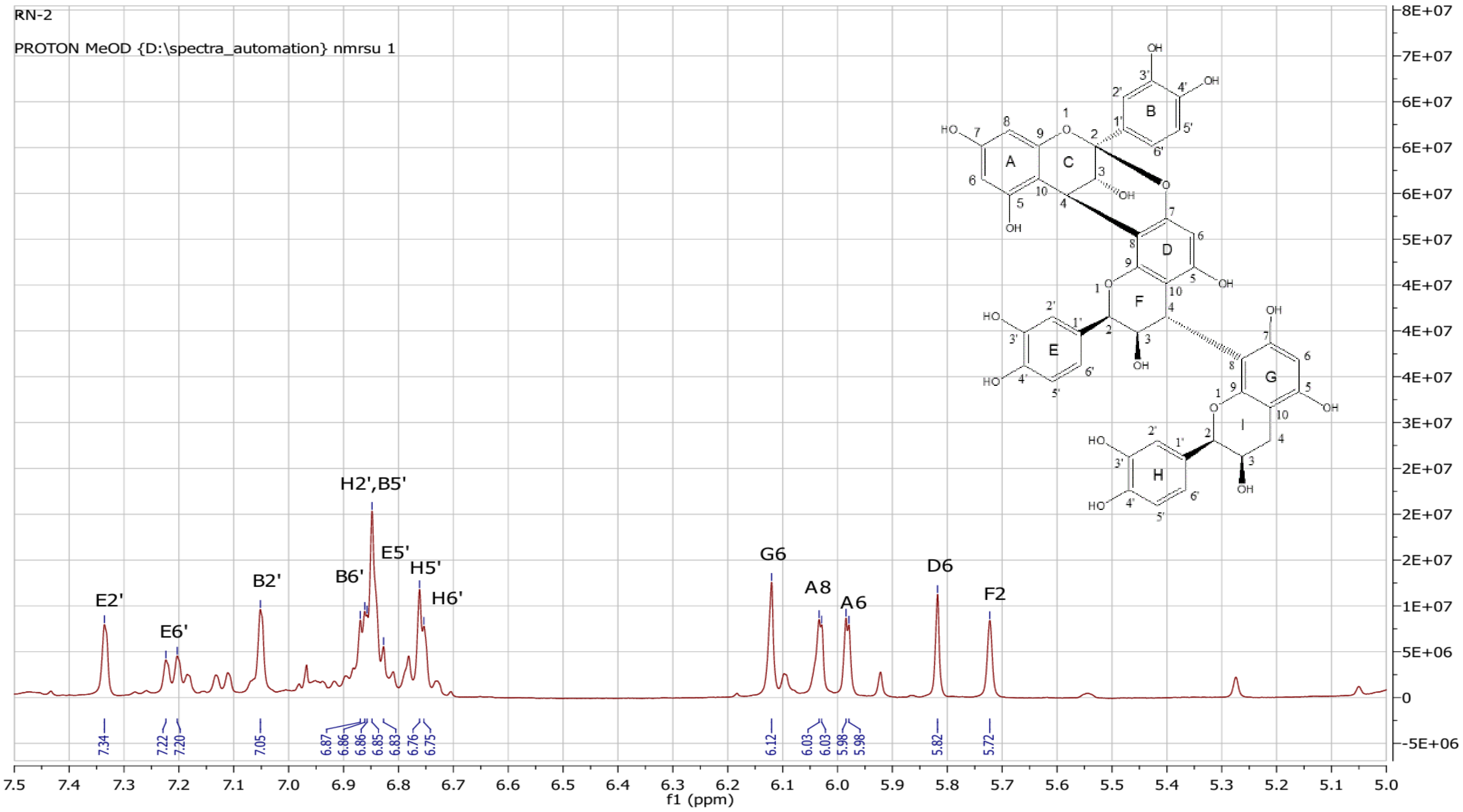

Figure S4. ^{1}H-NMR spectrum of Cinnamtannin B1 (RN2) (CD_3OD, 400 MHz)

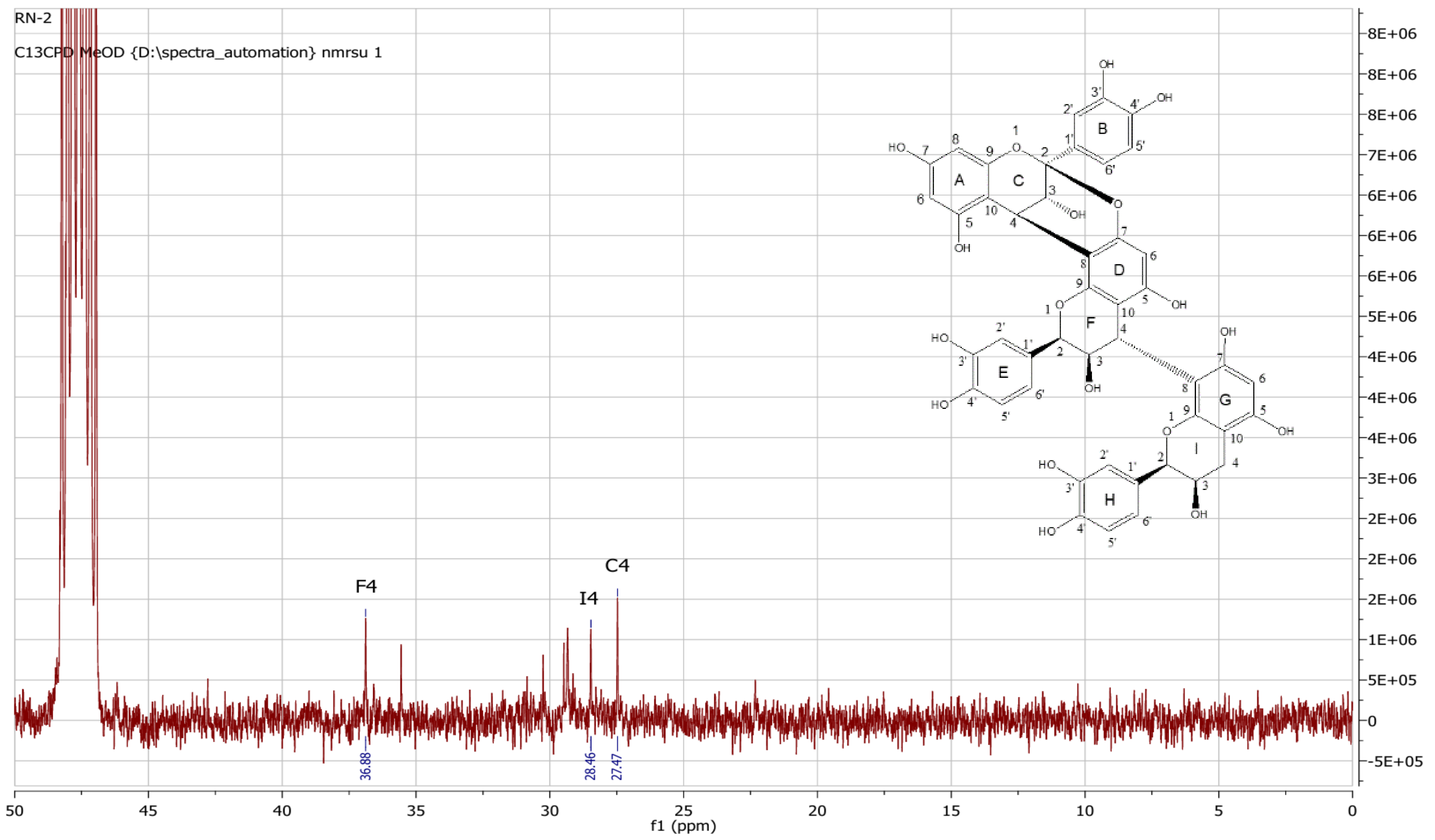

Figure S5. ^{13}C-NMR spectrum of Cinnamtannin B1 (RN2) (CD_3OD, 100 MHz)

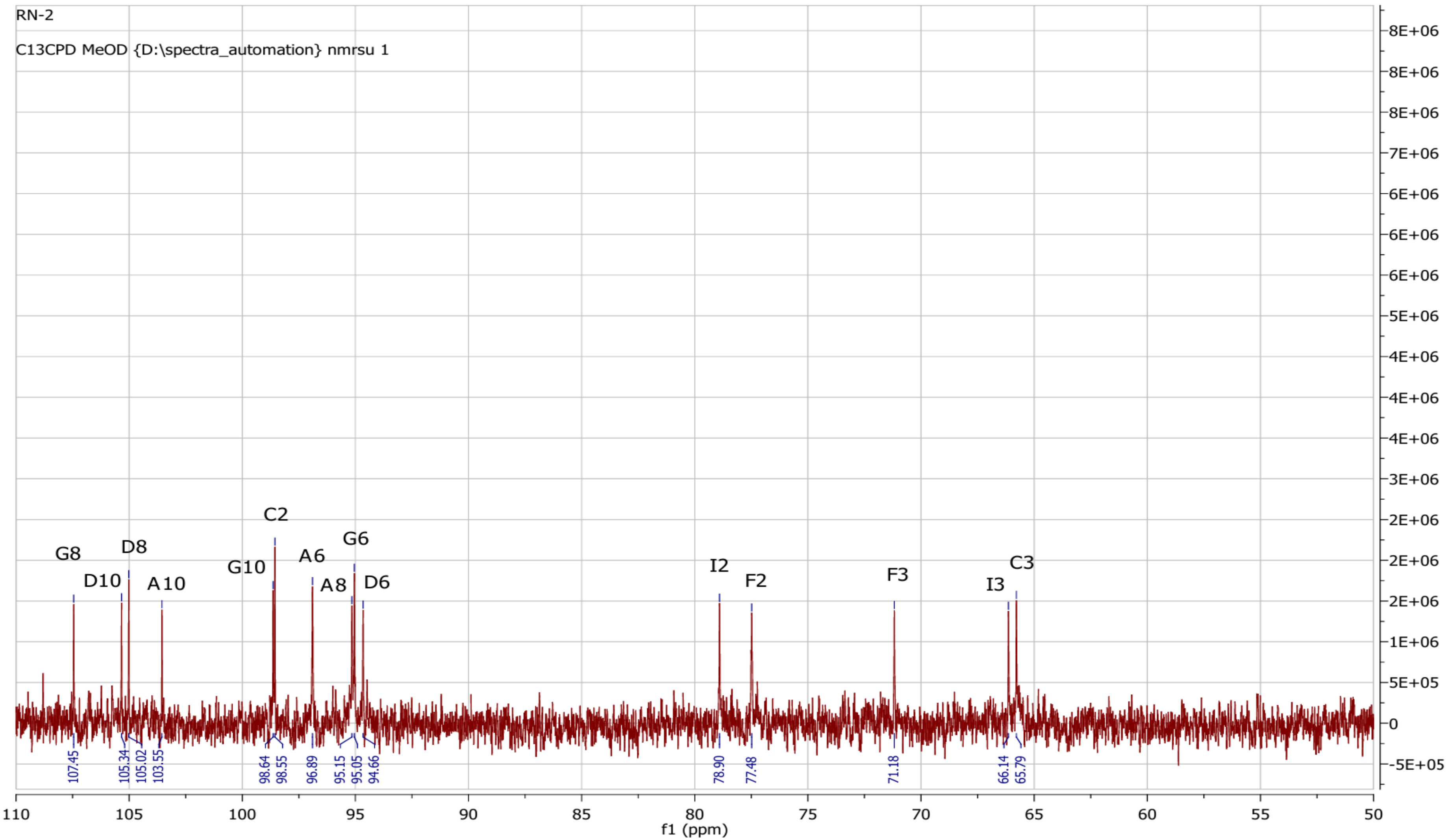

Figure S6. ^{13}C-NMR spectrum of Cinnamtannin B1 (RN2) (CD_3OD, 100 MHz)

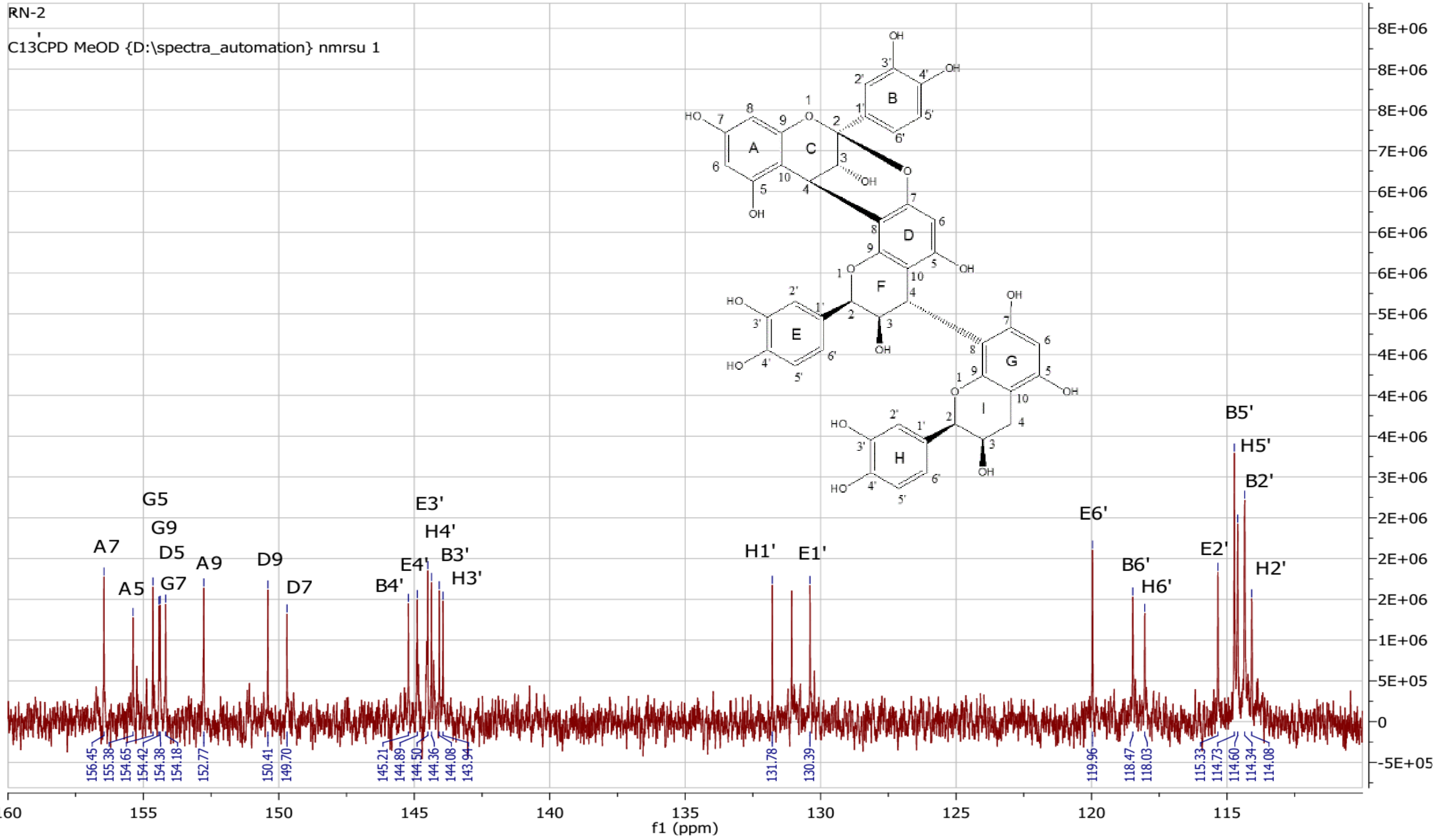

Figure S7. ^{13}C-NMR spectrum of Cinnamtannin B1 (RN2) (CD_3OD, 100 MHz)

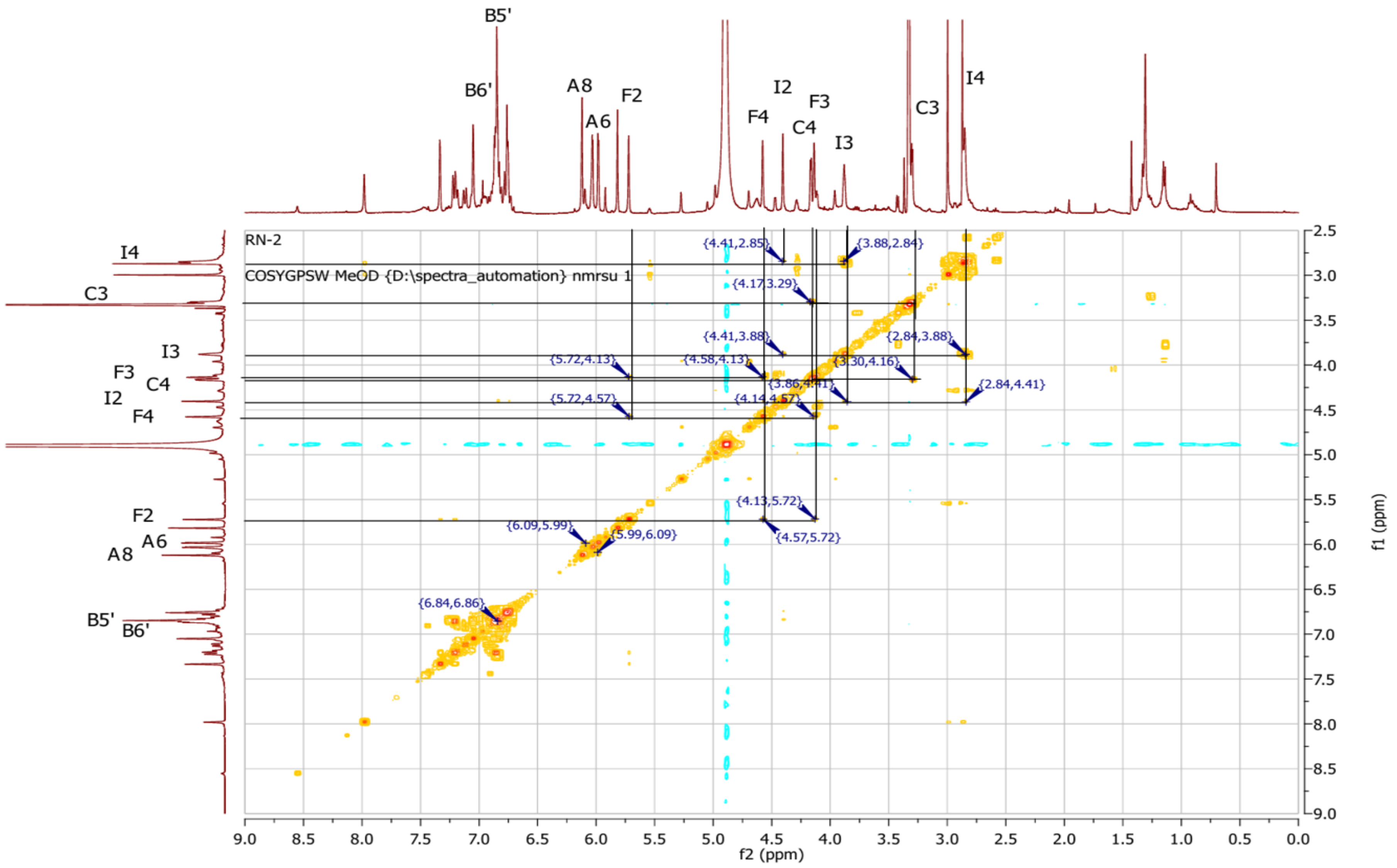

Figure S8. COSY spectrum of Cinnamtannin B1 (RN2)

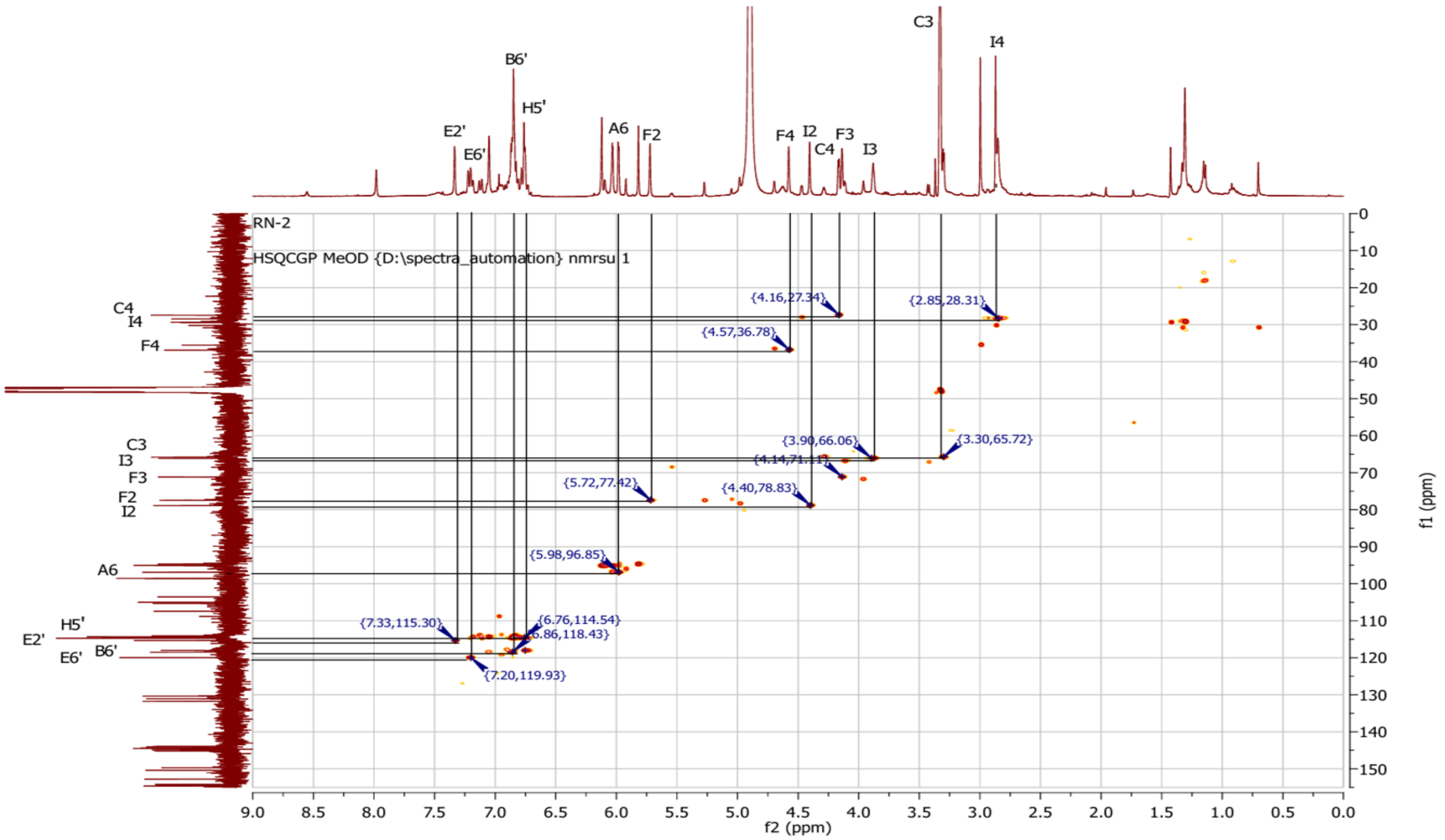

Figure S9. HSQC spectrum of the Cinnamtannin B1 (RN2)

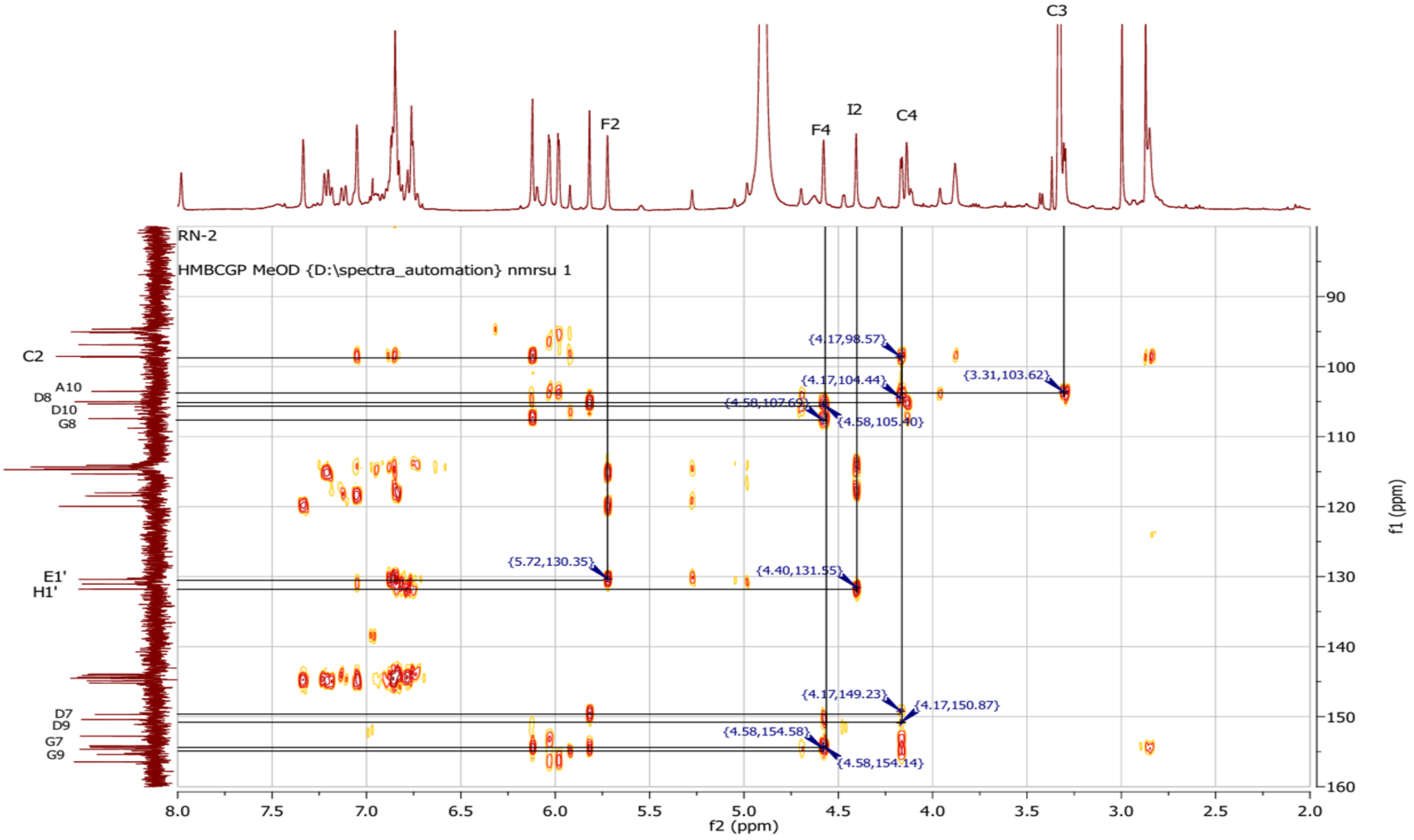

Figure S10. HMBC spectrum of the Cinnamtannin B1 (RN2)

Table S1. Assignments of ^{1}H NMR and ^{13}C NMR signals for Cinnamtannin B1 (RN2)

C/H Atom		δC ppm	δH ppm	*J* (Hz)	HMBC (H→C)
**Unit I**					
**C Ring**					
2	C	98.55	-		
3	CH	65.79	3.3	d (3.4)	A10
4	CH	27.47	4.17	d (3.4)	C2, D7, D8, D9
**A Ring**					
5	C	155.38	-		
6	CH	96.89	5.98	d (2.1)	A5, A7, A8, A10
7	C	156.45	-		
8	CH	95.15	6.03	d (2.1)	A6, A7, A10
9	C	152.77	-		
10	C	103.55	-		
**B Ring**					
1'	C	132.44	-		
2'	CH	114.34	7.05	gs	A2, B3', B4', B6'
3'	C	144.08	-		
4'	C	145.21	-		
5'	CH	114.73	6.84	d (8.4)	B3'
6'	CH	118.47	6.86	d (8.4)	B4'
**Unit II**					
**F Ring**					
2	CH	77.48	5.72	gs	E1', E2', E6'
3	CH	71.18	4.14	s	F2, F4, D10
4	CH	36.88	4.58	s	F2, F3, D10, G7, G8, G9
**D Ring**					
5	C	154.38	-		
6	CH	94.66	5.82	s	D5, D7, D8
7	C	149.7	-		
8	C	105.02	-		
9	C	150.41	-		
10	C	105.34	-		

Table S1. Assignments of ^{1}H NMR and ^{13}C NMR signals for Cinnamtannin B1 (RN2)

C/H Atom		δC ppm	δH ppm	*J* (Hz)	HMBC (H→C)
**E Ring**					
1'	C	130.39	-		
2'	CH	115.33	7.34	gs	E3', E4', E6', F2
3'	C	144.5	-		
4'	C	144.89	-		
5'	CH	114.35	6.83	*	E1', E3', E4', E6'
6'	CH	119.96	7.21	d (8.2)	E2', E4', F2
**Unit III**					
**I Ring**					
2	CH	78.9	4.41	s	H1', H2', H6', I3
3	CH	66.14	3.88	gs	-
4	CH_2	28.46	2.85	*	G9, G10, I2, I3
**G Ring**					
5	C	154.65	-		
6	CH	95.05	6.12	s	G7, G8, G10
7	C	154.18	-		
8	C	107.45	-		
9	C	154.42	-		
10	C	98.64	-		
**H Ring**					
1'	C	131.78	-		
2'	CH	114.08	6.84	*	H1', H3', H4', H6'
3'	C	143.94	-		
4'	C	144.36	-		
5'	CH	114.6	6.76	*	H1', H3'
6'	CH	118.03	6.75	*	H1', H2', H5'

*Could not be solved due to interference

(-)-Epicatechin Gallate (RN3)

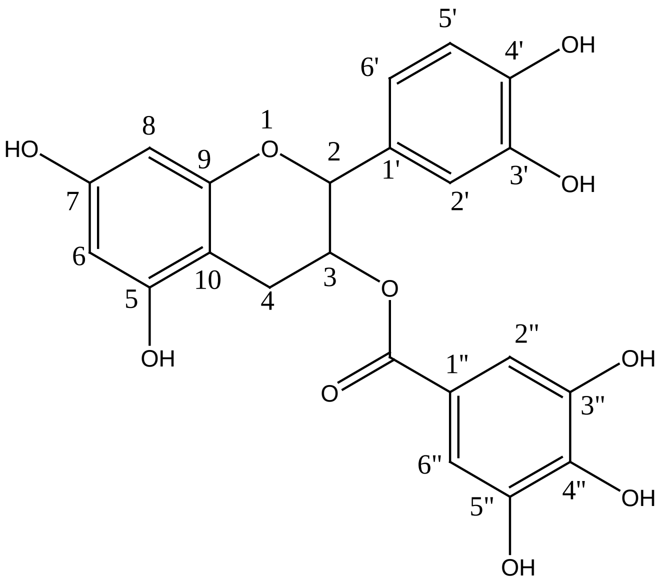

Figure	Caption	Page
**Figure S11**	HR-ESI-MS spectrum of (-)-Epicatechin gallate (RN3)	15
**Figure S12**	^{1}H-NMR spectrum of (-)-Epicatechin gallate (RN3)	16
**Figure S13**	^{13}C-NMR spectrum of (-)-Epicatechin gallate (RN3)	17
**Figure S14**	COSY spectrum of (-)-Epicatechin gallate (RN3)	18
**Figure S15**	HSQC spectrum of (-)-Epicatechin gallate (RN3)	19
**Figure S16**	HMBC spectrum of (-)-Epicatechin gallate (RN3)	20
**Table S2**	Assignments of 1H NMR and 13C NMR signals for (-)-Epicatechin gallate (RN3)	21

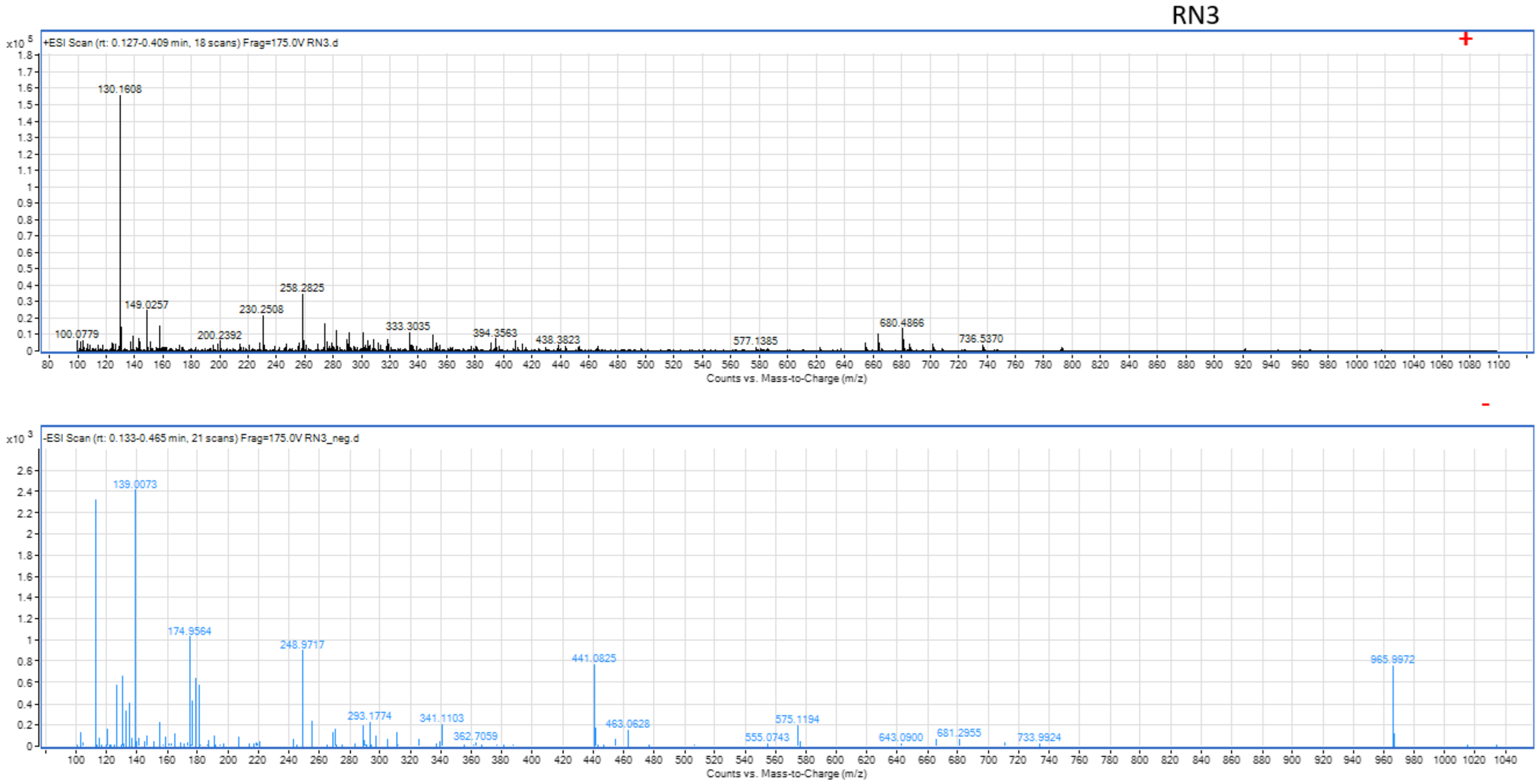

Figure S11. HR-ESI-MS spectrum of (-)-Epicatechin gallate (RN3): (a) +ESI mass spectrum, (b) –ESI mass spectrum, (c) Molecular ion peak

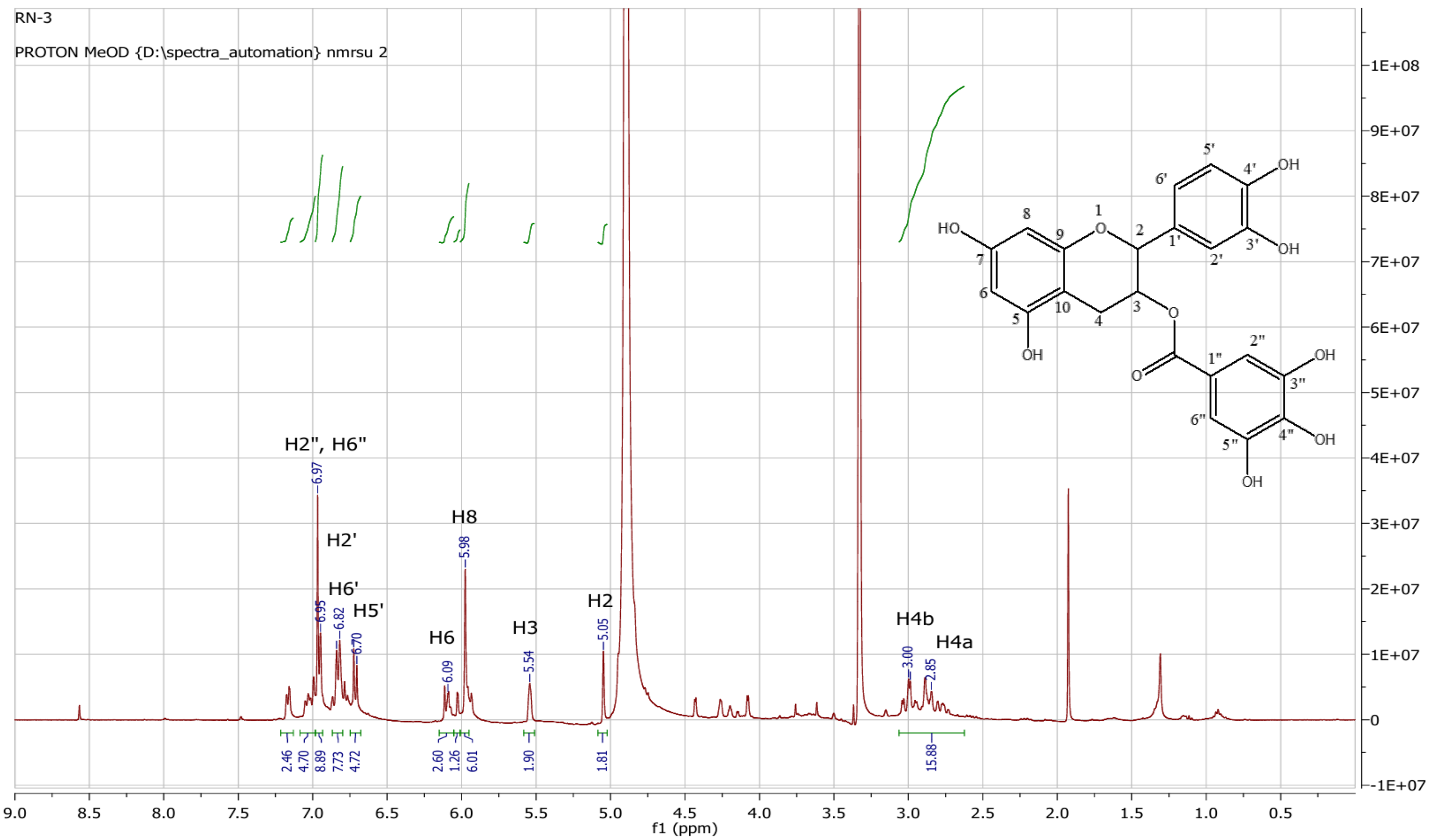

Figure S12. ^{1}H-NMR spectrum of (-)-Epicatechin gallate (RN3) (CD_3OD, 400 MHz)

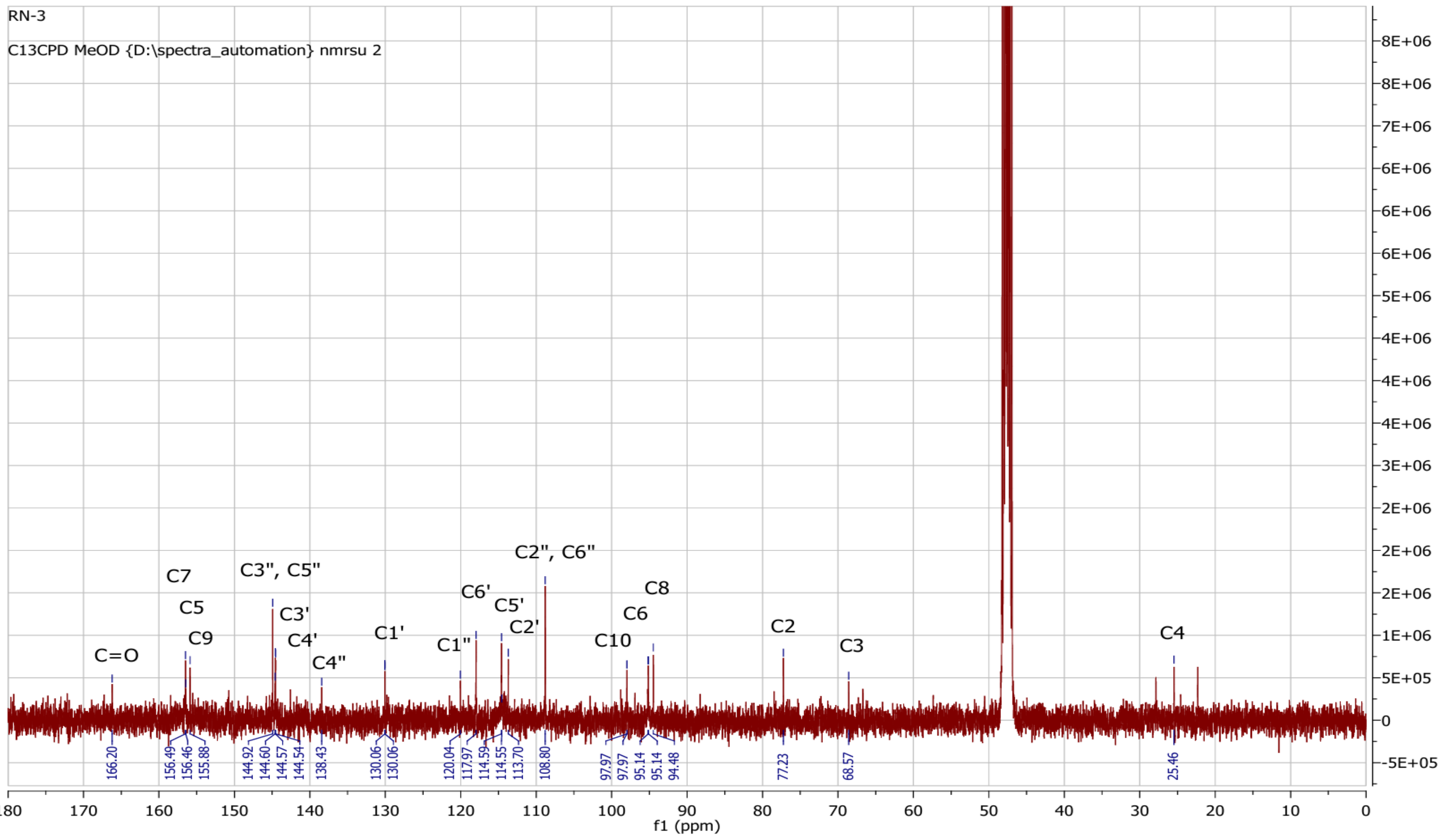

Figure S13. ^{13}C-NMR spectrum of (-)-Epicatechin gallate (RN3) (CD_3OD, 100 MHz)

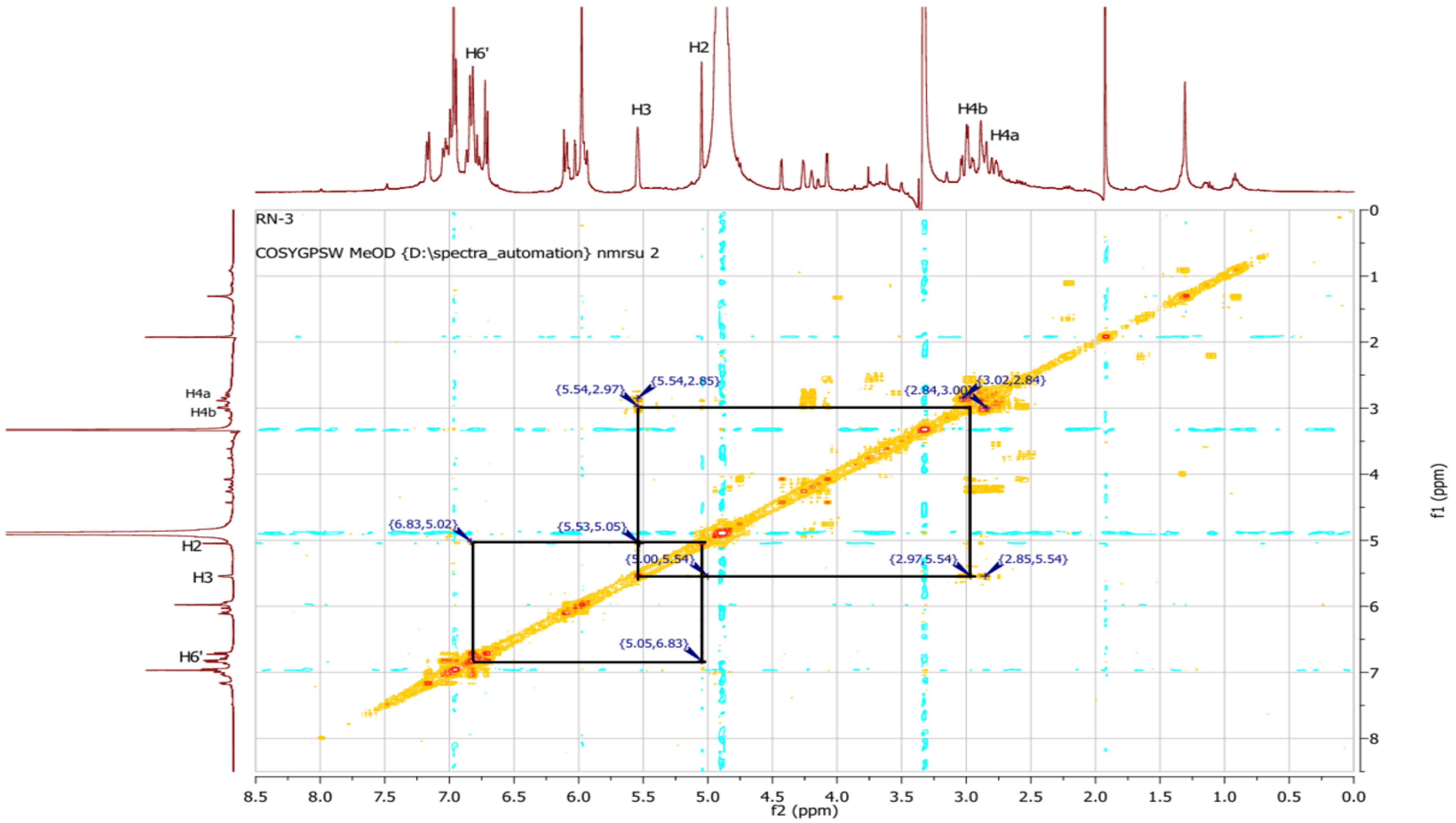

Figure S14. COSY spectrum of (-)-Epicatechin gallate (RN3)

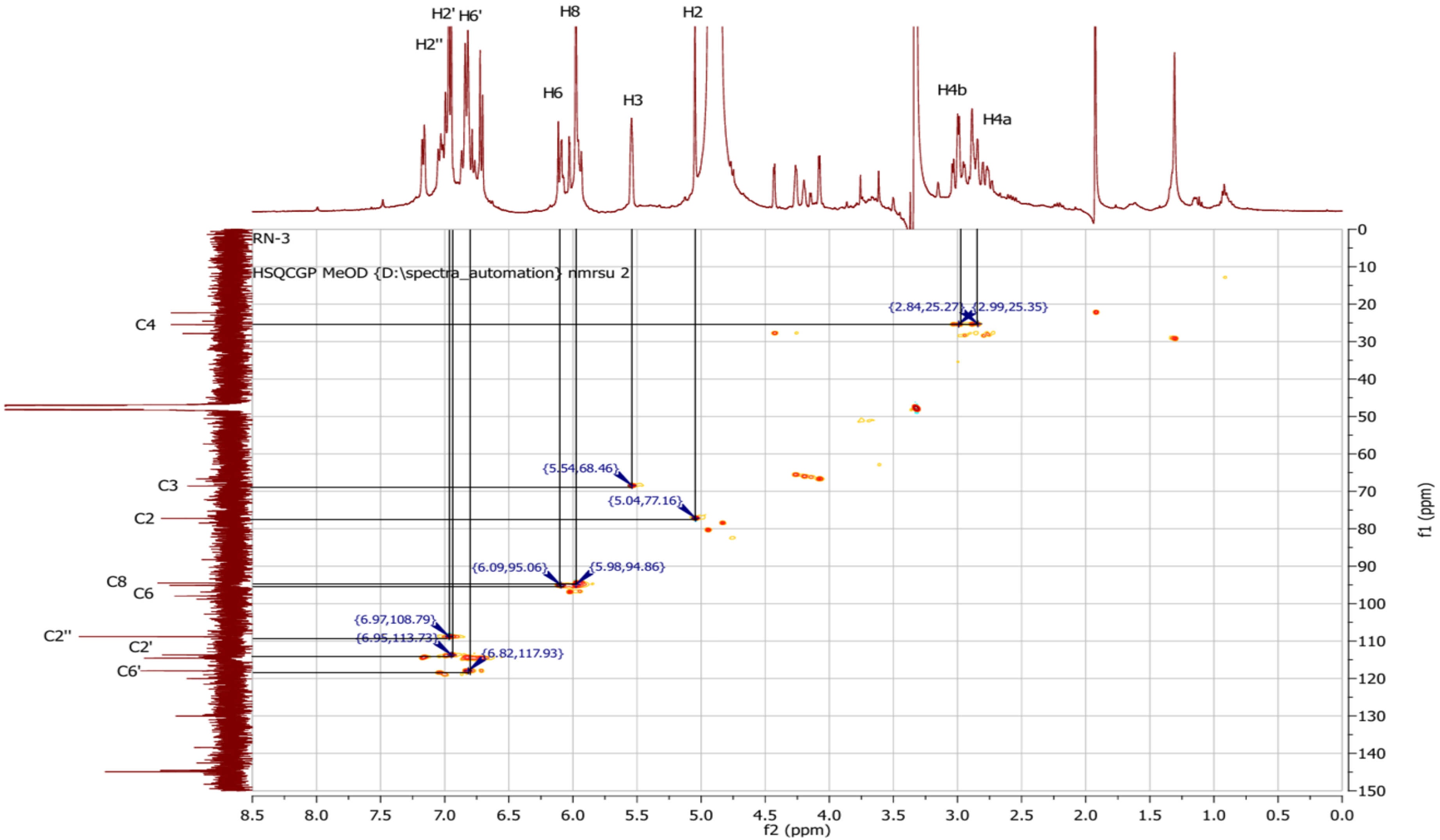

Figure S15. HSQC spectrum of (-)-Epicatechin gallate (RN3)

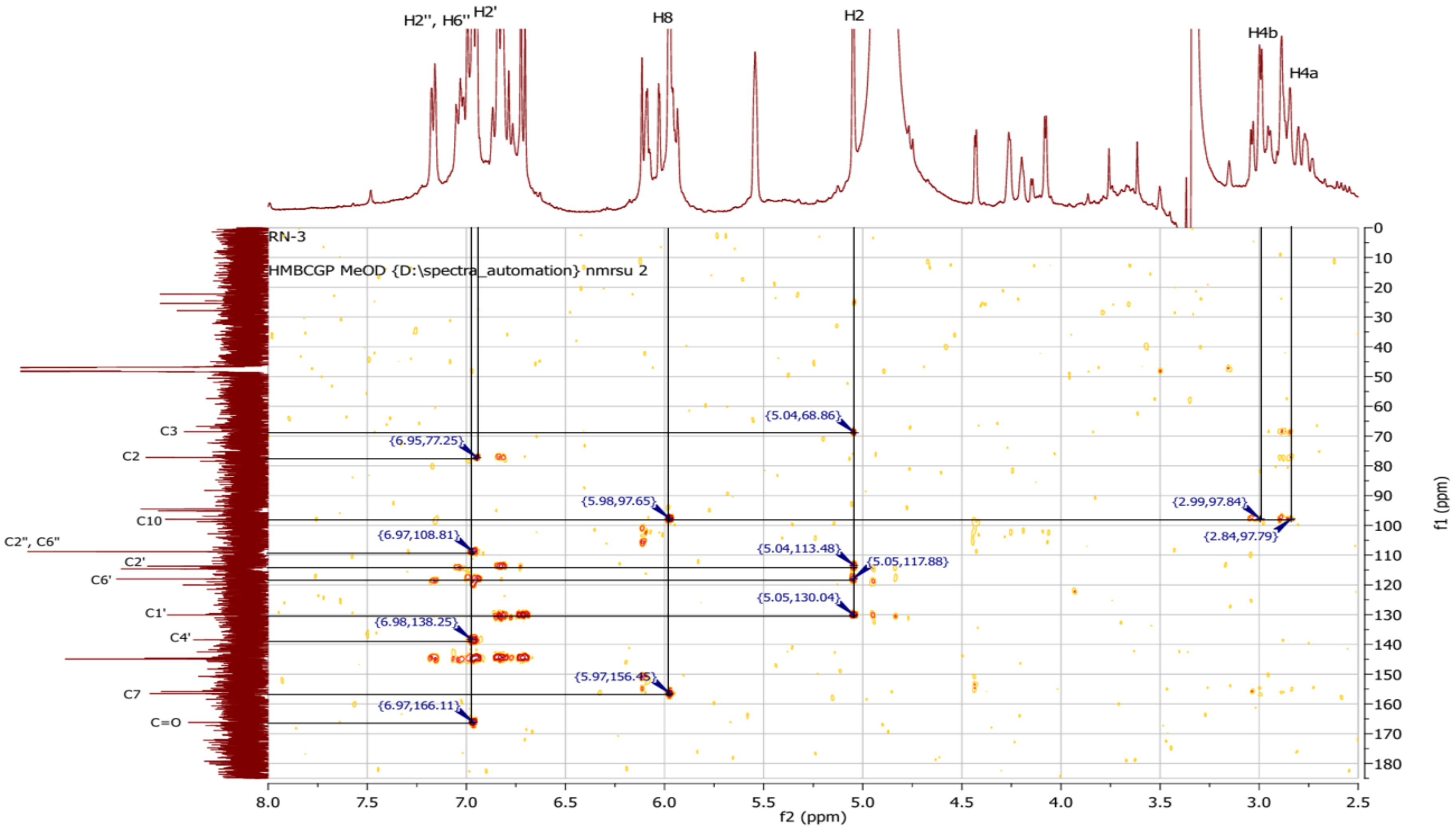

Figure S16. HMBC spectrum of (-)-Epicatechin gallate (RN3)

Table S2. Assignments of ^{1}H NMR and ^{13}C NMR signals for (-)-Epicatechin gallate (RN3)

C/H Atom		δC ppm	δH ppm	*J* (Hz)	HMBC (H→C)
2	CH	77.23	5.04	s	3, 1', 2', 6'
3	CH	68.57	5.54	m	
4	CH_2	25.46	2.84	dd (17.3/2.4)	10
			2.99	dd (17.3/4.7)	
5	C	156.46	-		
6	CH	95.14	6.09	d (2.3)	
7	C	156.49	-		
8	CH	94.48	5.98	d (2.3)	7, 10
9	C	155.88	-		
10	C	97.97	-		
1'	C	130.06	-		
2'	CH	113.70	6.95	d (2.0)	2
3'	C	144.57	-		
4'	C	144.54	-		
5'	C	114.56	6.71	d (8.2)	1',4'
6'	CH	117.94	6.81	*	
**R (Gallic acid)**					
1"	C	120.04	-		
2"	CH	108.8	6.97	s	CO
3"	C	144.92	-		
4"	C	138.43	-		
5"	C	144.92	-		
6"	CH	108.80	6.97	s	CO
CO	CO	166.20	-		

*Could not be solved due to interference
